# Supplementary material for: Polyketide synthase gene clusters in the ericoid mycorrhizal fungus Oidiodendron maius: genome-wide prediction, expression profiling, and functional characterization of knockout mutants
Source: Front Plant Sci. 2026 Sep 16;17:1924430. doi: 10.3389/fpls.2026.1924430 (PMC13623903; doi:10.3389/fpls.2026.1924430)
Supplement: Supplementary file 1 [file SupplementaryFile1.zip › Supplementary Materials/Supplementary Folder 1.zip!/Oidma1_AssemblyScaffolds_Repeatmasked/knownclusterblast/cluster31/jgi.p_Oidma1_203951_mibig_hits.html]

| MIBiG Protein | Description | MIBiG Cluster | MiBiG Product | % ID | % Coverage | BLAST Score | E-value |
| --- | --- | --- | --- | --- | --- | --- | --- |
| CBF87069.1 | nonribosomal\_peptide\_synthase,\_putative\_(Eurofung) | BGC0001290 | Emericellamide\_A\_/\_emericellamide\_B\_biosynthetic\_gene\_cluster | 32 | 100.859220093 | 788 | 1.6e-227 |
| ctg1\_orf003 |  | BGC0000334 | Cyclosporin\_biosynthetic\_gene\_cluster | 32 | 95.8360872439 | 707 | 3.7e-203 |
| AFT91378.1 | EcdA | BGC0000338 | Echinocandin\_B\_biosynthetic\_gene\_cluster | 32 | 101.520158625 | 683 | 7.4e-196 |
| CAP93139.1 |  | BGC0000357 | Fungisporin\_biosynthetic\_gene\_cluster | 35 | 72.8354263054 | 666 | 5.5e-191 |
| BAN91483.1 | putative\_Aculeacin\_A\_nonribosomal\_peptide\_synthase | BGC0001220 | Aculeacin\_A\_biosynthetic\_gene\_cluster | 30 | 104.560475876 | 665 | 1.6e-190 |
| AMM63162.1 | AniA | BGC0001371 | Echinocandin\_B\_biosynthetic\_gene\_cluster | 32 | 93.2584269663 | 661 | 1.8e-189 |
| EPE34341.1 | Acetyl-CoA\_synthetase-like\_protein | BGC0001035 | Pneumocandin\_biosynthetic\_gene\_cluster | 31 | 95.7699933906 | 655 | 1.7e-187 |
| BAO73252.1 | non-ribosomal\_peptide\_synthetase | BGC0000456 | Verlamelin\_biosynthetic\_gene\_cluster | 31 | 94.4481163252 | 651 | 3.1e-186 |
| OAQ83772.1 | nonribosomal\_peptide\_synthase | BGC0001358 | Leucinostatins\_biosynthetic\_gene\_cluster | 30 | 97.9510905486 | 650 | 5.3e-186 |
| AAF01762.1 | AM-toxin\_synthetase | BGC0001261 | AM-toxin\_biosynthetic\_gene\_cluster | 31 | 93.9854593523 | 624 | 3.1e-178 |
| EFY94500.1 | non-ribosomal\_peptide\_synthetase | BGC0000337 | Destruxins\_biosynthetic\_gene\_cluster | 34 | 70.323859881 | 614 | 4.2e-175 |
| EAL89049.1 | nonribosomal\_peptide\_synthase,\_putative | BGC0000355 | Fumiquinazolines\_biosynthetic\_gene\_cluster | 34 | 70.4560475876 | 589 | 1.5e-167 |
| CRG85572.1 | nonribosomal\_peptide\_synthase,\_putative | BGC0001402 | Cyclochlorotine\_biosynthetic\_gene\_cluster | 29 | 97.5545274289 | 586 | 7.2e-167 |
| ADM34138.1 | non-ribosomal\_peptide\_synthetase | BGC0001084 | Notoamide\_/\_stephacidin\_biosynthetic\_gene\_cluster | 29 | 96.0343688037 | 583 | 6.1e-166 |
| CAP96445.1 |  | BGC0000420 | Roquefortine\_C\_/\_meleagrin\_biosynthetic\_gene\_cluster | 28 | 102.115003305 | 576 | 7.5e-164 |
| AGC83576.1 | NRPS | BGC0000818 | Notoamide\_biosynthetic\_gene\_cluster | 28 | 95.7699933906 | 574 | 3.7e-163 |
| ACZ66258.1 | APS1 | BGC0000304 | Apicidin\_biosynthetic\_gene\_cluster | 30 | 94.2498347654 | 559 | 1.6e-158 |
| CBF76038.1 | nonribosomal\_peptide\_synthase,\_putative\_(Eurofung) | BGC0001399 | Fellutamide\_B\_biosynthetic\_gene\_cluster | 33 | 71.5796430932 | 555 | 1.8e-157 |
| ABB90279.1 | non-ribosomal\_peptide\_synthetase | BGC0001057 | Zearalenone\_biosynthetic\_gene\_cluster | 33 | 72.504957039 | 551 | 2.6e-156 |
| EAW16180.1 | nonribosomal\_peptide\_synthase,\_putative | BGC0000293 | Acetylaszonalenin\_biosynthetic\_gene\_cluster | 31 | 70.8526107072 | 544 | 4.1e-154 |
| BAH23995.1 | nonribosomal\_peptide\_synthetase | BGC0000356 | Fumitremorgin\_biosynthetic\_gene\_cluster | 30 | 93.5888962327 | 534 | 3.3e-151 |
| AGQ43600.1 | HC-toxin\_synthetase | BGC0001166 | HC-toxin\_biosynthetic\_gene\_cluster | 29 | 97.6206212822 | 529 | 1e-149 |
| ADY16697.1 | TqaA | BGC0001142 | Tryptoquialanine\_biosynthetic\_gene\_cluster | 32 | 72.8354263054 | 524 | 5.7e-148 |
| ABM91454.1 | lysergyl\_peptide\_synthetase\_LpsB | BGC0000348 | Ergovaline\_biosynthetic\_gene\_cluster | 31 | 80.9649702578 | 508 | 2.5e-143 |
| XP\_001227984.1 | hypothetical\_protein | BGC0001240 | Serinocyclin\_A\_/\_serinocyclin\_B\_biosynthetic\_gene\_cluster | 29 | 96.100462657 | 506 | 1.2e-142 |
| AAA33023.2 | HC-toxin\_synthetase | BGC0000370 | HC-toxin\_biosynthetic\_gene\_cluster | 32 | 73.6946463979 | 504 | 3.6e-142 |
| AGA37267.1 | NRPS | BGC0000816 | Malbrancheamide\_biosynthetic\_gene\_cluster | 31 | 72.1744877726 | 495 | 1.7e-139 |
| ACR78148.1 | BSLS | BGC0000312 | Bassianolide\_biosynthetic\_gene\_cluster | 28 | 87.4421678784 | 493 | 1.1e-138 |
| ACI30655.1 | BEAS\_beauvericin\_nonribosomal\_cyclodepsipeptide\_synthetase | BGC0000313 | Beauvericin\_biosynthetic\_gene\_cluster | 28 | 89.6893588896 | 470 | 5.8e-132 |
| CAA79245.2 | enniatin\_synthetase | BGC0000342 | Enniatin\_biosynthetic\_gene\_cluster | 30 | 72.5710508923 | 464 | 4.1e-130 |
| ctg1\_orf00001 |  | BGC0000901 | Ferrichrome\_biosynthetic\_gene\_cluster | 26 | 97.9510905486 | 464 | 5.4e-130 |
| ctg1\_orf000000 |  | BGC0000900 | Ferrichrome\_biosynthetic\_gene\_cluster | 31 | 63.7144745539 | 434 | 4.6e-121 |
| AGA37269.1 | NRPS | BGC0000819 | Paraherquamide\_biosynthetic\_gene\_cluster | 29 | 65.2346331791 | 426 | 1.2e-118 |
| CAI59267.1 | peptide\_synthetase\_3 | BGC0001263\_c2 | Ergometrine\_biosynthetic\_gene\_cluster | 29 | 72.504957039 | 423 | 1.4e-117 |
| ADY16689.1 | TqaB | BGC0001142 | Tryptoquialanine\_biosynthetic\_gene\_cluster | 30 | 63.3179114342 | 416 | 1.3e-115 |
| AFN69082.1 | nonribosomal\_peptide\_synthetase\_6 | BGC0001249 | Dimethylcoprogen\_biosynthetic\_gene\_cluster | 29 | 68.3410442829 | 409 | 1.6e-113 |
| CAD29798.1 | peptide\_synthetase | BGC0001015 | Microcystin\_biosynthetic\_gene\_cluster | 26 | 87.5082617317 | 391 | 3.4e-108 |
| BAH43871.1 | truncated\_linear\_pentadecapeptide\_gramicidin\_synthetase\_LgrC | BGC0000367 | Gramicidin\_biosynthetic\_gene\_cluster | 26 | 86.384666226 | 388 | 2.9e-107 |
| EAL89046.1 | nonribosomal\_peptide\_synthase,\_putative | BGC0000355 | Fumiquinazolines\_biosynthetic\_gene\_cluster | 30 | 67.7461996034 | 388 | 3.8e-107 |
| CAQ71829.1 | non\_ribosomal\_peptide\_synthase,\_antibiotic\_synthesis;\_contains\_4\_condensation\_domains,\_3\_AMP-acid\_ligases\_II\_domains,\_3\_PP-binding,\_Phosphopantetheine\_attachment\_site\_and\_a\_putative\_thioesterase\_domain | BGC0001189 | Taiwachelin\_biosynthetic\_gene\_cluster | 27 | 103.767349636 | 387 | 8.4e-107 |
| AHF21228.1 | TriD | BGC0000449 | Tridecaptin\_biosynthetic\_gene\_cluster | 24 | 103.701255783 | 387 | 8.4e-107 |
| ANY57892.1 | PenN | BGC0001372 | Penigequinolone\_biosynthetic\_gene\_cluster | 30 | 63.9127561137 | 376 | 1.5e-103 |
| AHZ20773.1 | non-ribosomal\_peptide\_synthase | BGC0000369 | Hassallidins\_biosynthetic\_gene\_cluster | 28 | 71.5796430932 | 375 | 3.3e-103 |
| AEH41794.1 | HrmP | BGC0000374 | Hormaomycin\_biosynthetic\_gene\_cluster | 28 | 73.4302709848 | 374 | 7.4e-103 |
| CAJ46692.1 | non\_ribosomal\_peptide\_synthase | BGC0000969 | Chondramide\_biosynthetic\_gene\_cluster | 28 | 72.0423000661 | 372 | 2.8e-102 |
| AGM16413.1 | paenibacterin\_synthetase\_B | BGC0000400 | Paenibacterin\_biosynthetic\_gene\_cluster | 24 | 103.23859881 | 367 | 6.9e-101 |
| AHZ20774.1 | non-ribosomal\_peptide\_synthase | BGC0000369 | Hassallidins\_biosynthetic\_gene\_cluster | 26 | 87.7726371447 | 367 | 9e-101 |
| AIW82285.1 | PuwH | BGC0001125 | Puwainaphycins\_biosynthetic\_gene\_cluster | 28 | 73.1658955717 | 365 | 2.6e-100 |
| AHJ31215.1 | Long-chain-fatty-acid--CoA\_ligase | BGC0000430 | Spumigin\_biosynthetic\_gene\_cluster | 28 | 70.4560475876 | 363 | 9.9e-100 |
| ALK27914.1 | non-ribosomal\_peptide\_synthase | BGC0001233 | Feglymycin\_biosynthetic\_gene\_cluster | 28 | 71.2491738268 | 361 | 6.4e-99 |
| ACJ04424.1 | aureobasidin\_A1\_biosynthesis\_complex | BGC0000307 | Aureobasidin\_A1\_biosynthetic\_gene\_cluster | 29 | 59.4844679445 | 359 | 1.4e-98 |
| AAZ03552.1 | McnC | BGC0000332 | Cyanopeptolin\_biosynthetic\_gene\_cluster | 26 | 87.0456047588 | 358 | 4.2e-98 |
| AIG79241.1 | Hypothetical\_protein | BGC0000419 | Ristomycin\_A\_biosynthetic\_gene\_cluster | 29 | 71.3813615334 | 355 | 3.5e-97 |
| ABV79986.1 | ApnB | BGC0000301 | Anabaenopeptin\_biosynthetic\_gene\_cluster | 27 | 74.4216787839 | 354 | 6e-97 |
| AEU11003.1 | NpnC | BGC0001029 | Nostophycin\_biosynthetic\_gene\_cluster | 28 | 73.8268341044 | 353 | 1e-96 |
| AGM16412.1 | paenibacterin\_synthetase\_A | BGC0000400 | Paenibacterin\_biosynthetic\_gene\_cluster | 24 | 103.106411104 | 353 | 1.3e-96 |
| AIE77059.1 | peptide\_synthetase | BGC0000418 | Ristocetin\_biosynthetic\_gene\_cluster | 28 | 73.4963648381 | 351 | 5.1e-96 |
| ADG27359.1 | peptide\_synthetase | BGC0000296 | Actinomycin\_biosynthetic\_gene\_cluster | 27 | 86.1863846662 | 349 | 1.9e-95 |
| ADJ63842.1 | non-ribosomal\_peptide\_synthetase\_protein | BGC0000424 | Serobactins\_biosynthetic\_gene\_cluster | 28 | 74.4216787839 | 348 | 4.3e-95 |
| CAJ77715.1 | Mps1\_protein | BGC0000364 | Glycopeptidolipid\_biosynthetic\_gene\_cluster | 27 | 72.3727693325 | 347 | 5.6e-95 |
| AEA30274.1 | peptide\_synthetase | BGC0000429 | Skyllamycin\_biosynthetic\_gene\_cluster | 29 | 64.0449438202 | 347 | 5.6e-95 |
| AEH41793.1 | HrmO | BGC0000374 | Hormaomycin\_biosynthetic\_gene\_cluster | 29 | 63.7144745539 | 347 | 9.6e-95 |
| AAO23334.1 | NcpB | BGC0000397 | Nostocyclopeptide\_biosynthetic\_gene\_cluster | 28 | 64.1771315268 | 346 | 1.3e-94 |
| AAT01806.1 | non-ribosomal\_peptide\_synthetase | BGC0000365 | Glycopeptidolipid\_biosynthetic\_gene\_cluster | 27 | 73.0337078652 | 345 | 3.7e-94 |
| CAA11795.1 | PCZA363.4 | BGC0000322 | Chloroeremomycin\_biosynthetic\_gene\_cluster | 29 | 64.8380700595 | 344 | 4.8e-94 |
| CAJ77695.1 | MPS1\_protein | BGC0000363 | Glycopeptidolipid\_biosynthetic\_gene\_cluster | 27 | 71.9101123596 | 344 | 8.2e-94 |
| AAY93356.2 | non-ribosomal\_peptide\_synthetase\_PvdI | BGC0000413 | Pyoverdine\_biosynthetic\_gene\_cluster | 27 | 72.3727693325 | 342 | 3.1e-93 |
| CAG15011.1 | peptide\_synthetase,\_module\_4-6 | BGC0000441 | Teicoplanin\_biosynthetic\_gene\_cluster | 28 | 73.0998017184 | 341 | 6.9e-93 |
| CAE53352.1 | non-ribosomal\_peptide\_synthetase | BGC0000440 | Teicoplanin\_biosynthetic\_gene\_cluster | 28 | 73.0998017184 | 341 | 6.9e-93 |
| ACZ55943.1 | non-ribosomal\_peptide\_synthetase | BGC0000302 | Anabaenopeptin\_biosynthetic\_gene\_cluster | 27 | 73.4963648381 | 340 | 9e-93 |
| AAY93354.1 | non-ribosomal\_peptide\_synthetase\_PvdD | BGC0000413 | Pyoverdine\_biosynthetic\_gene\_cluster | 27 | 73.0998017184 | 340 | 1.2e-92 |
| AHB82058.1 | non\_ribosomal\_peptide\_synthetase | BGC0001019 | Microsclerodermins\_biosynthetic\_gene\_cluster | 27 | 70.8526107072 | 339 | 1.5e-92 |
| AAF08797.1 | MycC | BGC0001103 | Mycosubtilin\_biosynthetic\_gene\_cluster | 26 | 72.9015201586 | 338 | 4.5e-92 |
| CAC48361.1 | peptide\_synthetase | BGC0000311 | Balhimycin\_biosynthetic\_gene\_cluster | 28 | 64.4415069399 | 337 | 7.6e-92 |
| AAF08796.1 | MycB | BGC0001103 | Mycosubtilin\_biosynthetic\_gene\_cluster | 26 | 72.3066754792 | 336 | 1.7e-91 |
| ABS74180.1 | BmyB | BGC0001090 | Bacillomycin\_biosynthetic\_gene\_cluster | 26 | 72.1744877726 | 336 | 1.7e-91 |
| AAD44233.1 | PstA | BGC0000362 | Glycopeptidolipid\_biosynthetic\_gene\_cluster | 28 | 62.5908790482 | 334 | 4.9e-91 |
| CAE15637.1 |  | BGC0001128 | Luminmide\_biosynthetic\_gene\_cluster | 26 | 92.1348314607 | 334 | 4.9e-91 |
| AIW82283.1 | PuwF | BGC0001125 | Puwainaphycins\_biosynthetic\_gene\_cluster | 27 | 74.1573033708 | 333 | 1.4e-90 |
| AHB82071.1 | non\_ribosomal\_peptide\_synthetase | BGC0001231 | Microsclerodermins\_biosynthetic\_gene\_cluster | 24 | 86.2524785195 | 331 | 7.1e-90 |
| AFP87549.1 | NrpS | BGC0001135 | Bicornutin\_biosynthetic\_gene\_cluster | 26 | 86.6490416391 | 330 | 9.3e-90 |
| EWS95122.1 | hypothetical\_protein | BGC0000306 | Arylomycin\_biosynthetic\_gene\_cluster | 27 | 74.2894910773 | 329 | 2.1e-89 |
| AEP18655.1 | WAPS2 | BGC0000461 | WAP-8294A2\_(lotilibcin)\_biosynthetic\_gene\_cluster | 27 | 75.8757435558 | 329 | 2.1e-89 |
| BAH43870.1 | putative\_linear\_pentadecapeptide\_gramicidin\_synthetase\_LgrB | BGC0000367 | Gramicidin\_biosynthetic\_gene\_cluster | 27 | 73.4963648381 | 328 | 4.6e-89 |
| CAM59606.1 | non-ribosomal\_peptide\_synthetase | BGC0000297 | Aeruginoside\_biosynthetic\_gene\_cluster | 27 | 73.231989425 | 328 | 4.6e-89 |
| BAB69699.1 | iturin\_A\_synthetase\_B | BGC0001098 | Iturin\_biosynthetic\_gene\_cluster | 26 | 72.2405816259 | 328 | 4.6e-89 |
| WP\_020635019.1 | hypothetical\_protein | BGC0001211 | Albachelin\_biosynthetic\_gene\_cluster | 25 | 94.1176470588 | 327 | 6e-89 |
| AAM80537.1 | StaC | BGC0000290 | A47934\_biosynthetic\_gene\_cluster | 27 | 72.0423000661 | 327 | 1e-88 |
| ALK27915.1 | non-ribosomal\_peptide\_synthase | BGC0001233 | Feglymycin\_biosynthetic\_gene\_cluster | 28 | 65.2346331791 | 326 | 1.8e-88 |
| AAK81824.1 | peptide\_synthetase | BGC0000326 | Complestatin\_biosynthetic\_gene\_cluster | 29 | 62.7230667548 | 325 | 3e-88 |
| AEI58866.1 | peptide\_synthetase | BGC0000455 | Vancomycin\_biosynthetic\_gene\_cluster | 27 | 71.3152676801 | 324 | 5.1e-88 |
| CAD91212.1 | putative\_non-ribosomal\_peptide\_synthetase,\_modules\_4-6 | BGC0000289 | A40926\_biosynthetic\_gene\_cluster | 27 | 71.2491738268 | 322 | 2.5e-87 |
| AEI58867.1 | peptide\_synthetase | BGC0000455 | Vancomycin\_biosynthetic\_gene\_cluster | 28 | 72.1744877726 | 320 | 9.7e-87 |
| CAD17792.1 | probable\_non\_ribosomal\_peptide\_synthetase\_protein | BGC0001363 | Ralsolamycin\_biosynthetic\_gene\_cluster | 26 | 72.5710508923 | 320 | 9.7e-87 |
| AAK81826.1 | peptide\_synthetase | BGC0000326 | Complestatin\_biosynthetic\_gene\_cluster | 31 | 49.636483807 | 320 | 1.3e-86 |
| BAH43765.1 | tyrocidine\_synthetase\_II | BGC0000452 | Tyrocidine\_biosynthetic\_gene\_cluster | 26 | 74.6199603437 | 319 | 2.2e-86 |
| ABD65958.1 | nonribosomal\_peptide\_synthetase | BGC0000341 | Enduracidin\_biosynthetic\_gene\_cluster | 26 | 99.5373430271 | 319 | 2.2e-86 |
| AAY93445.1 | non-ribosomal\_peptide\_synthetase\_PvdL | BGC0000413 | Pyoverdine\_biosynthetic\_gene\_cluster | 28 | 79.1143423662 | 319 | 2.8e-86 |
| WP\_028678149.1 | non-ribosomal\_peptide\_synthetase | BGC0001228 | Retimycin\_biosynthetic\_gene\_cluster | 27 | 71.2491738268 | 319 | 2.8e-86 |
| ABX37382.1 | amino\_acid\_adenylation\_domain\_protein | BGC0000984 | Delftibactin\_biosynthetic\_gene\_cluster | 28 | 66.4904163913 | 319 | 2.8e-86 |
| AIE77057.1 | peptide\_synthetase | BGC0000418 | Ristocetin\_biosynthetic\_gene\_cluster | 27 | 71.9101123596 | 317 | 8.2e-86 |
| AGS77309.1 | NRPS\_modules\_4-6 | BGC0001178 | UK-68,597\_biosynthetic\_gene\_cluster | 27 | 70.9847984137 | 317 | 1.1e-85 |
| AAC06346.1 | bacitracin\_synthetase\_1 | BGC0000310 | Bacitracin\_biosynthetic\_gene\_cluster | 24 | 73.0337078652 | 316 | 1.4e-85 |
| AAM80539.1 | StaA | BGC0000290 | A47934\_biosynthetic\_gene\_cluster | 30 | 62.8552544613 | 316 | 2.4e-85 |
| CAE02631.1 | surfactin\_synthetase\_B | BGC0000433 | Surfactin\_biosynthetic\_gene\_cluster | 23 | 104.296100463 | 315 | 4.1e-85 |
| AAC06348.1 | bacitracin\_synthetase\_3 | BGC0000310 | Bacitracin\_biosynthetic\_gene\_cluster | 25 | 71.7118307997 | 314 | 5.3e-85 |
| AAO23333.1 | NcpA | BGC0000397 | Nostocyclopeptide\_biosynthetic\_gene\_cluster | 28 | 63.978849967 | 314 | 6.9e-85 |
| ACA97576.1 | PmxA | BGC0000408 | Polymyxin\_biosynthetic\_gene\_cluster | 23 | 104.428288169 | 314 | 6.9e-85 |
| CAC48362.1 | peptide\_synthetase | BGC0000311 | Balhimycin\_biosynthetic\_gene\_cluster | 27 | 73.231989425 | 313 | 1.2e-84 |
| AIW82277.1 | PuwA | BGC0001125 | Puwainaphycins\_biosynthetic\_gene\_cluster | 27 | 64.5736946464 | 313 | 1.2e-84 |
| AED90002.1 | non-ribosomal\_peptide\_synthetase\_ThaA | BGC0000443 | Thanamycin\_biosynthetic\_gene\_cluster | 26 | 88.1031064111 | 313 | 1.5e-84 |
| AAM80536.1 | StaD | BGC0000290 | A47934\_biosynthetic\_gene\_cluster | 27 | 74.4216787839 | 312 | 2.6e-84 |
| AEG64697.1 | LpmC | BGC0000379 | Laspartomycin\_biosynthetic\_gene\_cluster | 26 | 79.5109054858 | 312 | 2.6e-84 |
| WP\_010369428.1 | phenylalanine\_racemase | BGC0000314 | Bromoalterochromides\_biosynthetic\_gene\_cluster | 28 | 74.2894910773 | 312 | 3.4e-84 |
| AAU39360.1 | lichenysin\_synthase\_LchAB | BGC0000381 | Lichenysin\_biosynthetic\_gene\_cluster | 25 | 71.5135492399 | 310 | 1.3e-83 |
| AHD05614.1 | putative\_non-ribosomal\_peptide\_ligase/\_polyketide\_synthase\_hybrid | BGC0001033 | Paenilamicin\_biosynthetic\_gene\_cluster | 25 | 72.0423000661 | 309 | 1.7e-83 |
| AEW31022.1 | plipastatin\_synthetase | BGC0000407 | Plipastatin\_biosynthetic\_gene\_cluster | 26 | 76.8010575017 | 309 | 2.2e-83 |
| ACU36654.1 | amino\_acid\_adenylation\_domain\_protein | BGC0000392 | Mirubactin\_biosynthetic\_gene\_cluster | 27 | 74.0912095175 | 309 | 2.2e-83 |
| AOA33122.1 | nonribosomal\_peptide\_synthetase | BGC0001346 | Bananamides\_biosynthetic\_gene\_cluster | 26 | 79.3787177792 | 309 | 2.2e-83 |
| ABS74206.1 | FenD | BGC0001095 | Fengycin\_biosynthetic\_gene\_cluster | 23 | 102.577660278 | 309 | 2.2e-83 |
| AAF00960.1 | McyA | BGC0001017 | Microcystin\_biosynthetic\_gene\_cluster | 27 | 72.0423000661 | 308 | 3.8e-83 |
| ABQ96384.2 | fusaricidin\_synthetase | BGC0001152 | Fusaricidin\_biosynthetic\_gene\_cluster | 25 | 89.8215465962 | 308 | 5e-83 |
| AHH53506.1 | non-ribosomal\_peptide\_synthetase | BGC0000439 | Taromycin\_biosynthetic\_gene\_cluster | 29 | 64.7719762062 | 307 | 6.5e-83 |
| ALV86868.1 | Tlo22 | BGC0001406 | Telomycin\_biosynthetic\_gene\_cluster | 27 | 64.6397884997 | 307 | 6.5e-83 |
| ABI26077.1 | OciA | BGC0000331 | Cyanopeptin\_biosynthetic\_gene\_cluster | 25 | 71.777924653 | 307 | 8.5e-83 |
| AFH75320.1 | nonribosomal\_peptide\_synthetase | BGC0000425 | Sessilin\_biosynthetic\_gene\_cluster | 25 | 94.7785855915 | 307 | 8.5e-83 |
| EFE73313.1 | nonribosomal\_peptide\_synthetase | BGC0000431 | Stenothricin\_biosynthetic\_gene\_cluster | 28 | 73.0998017184 | 306 | 1.4e-82 |
| BAH43766.1 | tyrocidine\_synthetase\_III | BGC0000452 | Tyrocidine\_biosynthetic\_gene\_cluster | 24 | 95.1751487112 | 306 | 1.4e-82 |
| AED90003.1 | non-ribosomal\_peptide\_synthetase\_ThaB | BGC0000443 | Thanamycin\_biosynthetic\_gene\_cluster | 24 | 89.8215465962 | 306 | 1.4e-82 |
| AAO62586.1 | peptide\_sythetase | BGC0001016 | Microcystin\_biosynthetic\_gene\_cluster | 26 | 72.2405816259 | 306 | 1.9e-82 |
| ACZ55942.1 | non-ribosomal\_peptide\_synthetase | BGC0000302 | Anabaenopeptin\_biosynthetic\_gene\_cluster | 26 | 64.6397884997 | 305 | 2.5e-82 |
| AHI59109.1 | locillomycin\_synthase\_B | BGC0001005 | Locillomycin\_biosynthetic\_gene\_cluster | 26 | 72.9015201586 | 305 | 3.2e-82 |
| AEW31020.1 | plipastatin\_synthetase | BGC0000407 | Plipastatin\_biosynthetic\_gene\_cluster | 24 | 95.8360872439 | 305 | 4.2e-82 |
| AAZ23075.1 | peptide\_synthetase | BGC0000291 | A54145\_biosynthetic\_gene\_cluster | 28 | 66.8869795109 | 304 | 7.2e-82 |
| CBL93730.1 | NRPS | BGC0000360 | GE81112\_biosynthetic\_gene\_cluster | 30 | 65.3668208857 | 303 | 1.6e-81 |
| EFL06867.1 | predicted\_protein | BGC0000300 | Amychelin\_biosynthetic\_gene\_cluster | 29 | 63.9127561137 | 302 | 2.1e-81 |
| EFL06866.1 | predicted\_protein | BGC0000300 | Amychelin\_biosynthetic\_gene\_cluster | 26 | 72.7032385988 | 302 | 2.7e-81 |
| AJV88375.1 | MfnC | BGC0001214 | Marformycins\_biosynthetic\_gene\_cluster | 29 | 63.8466622604 | 302 | 3.6e-81 |
| CAM56770.1 |  | BGC0000354 | Friulimicin\_biosynthetic\_gene\_cluster | 26 | 86.9134170522 | 301 | 4.6e-81 |
| AHD05678.1 | nonribosomal\_peptide\_ligase\_subunit | BGC0000402 | Paenilarvins\_biosynthetic\_gene\_cluster | 27 | 55.2544613351 | 301 | 6.1e-81 |
| AIW82284.1 | PuwG | BGC0001125 | Puwainaphycins\_biosynthetic\_gene\_cluster | 26 | 75.9418374091 | 300 | 7.9e-81 |
| CAJ14039.1 | peptide\_synthetase\_PhsC | BGC0000406 | Phosphinothricin\_biosynthetic\_gene\_cluster | 28 | 64.0449438202 | 300 | 7.9e-81 |
| AAK81825.1 | peptide\_synthetase | BGC0000326 | Complestatin\_biosynthetic\_gene\_cluster | 28 | 64.8380700595 | 300 | 7.9e-81 |
| ALG65318.1 | Cal18 | BGC0001297 | WS9326\_biosynthetic\_gene\_cluster | 27 | 65.8294778586 | 300 | 7.9e-81 |
| CAY48788.1 | putative\_non-ribosomal\_peptide\_synthetase | BGC0001312 | Viscosin\_biosynthetic\_gene\_cluster | 25 | 95.3073364177 | 300 | 1e-80 |
| CAG29031.1 | nonribosomal\_peptide\_synthetase\_(modules\_1\_and\_2) | BGC0001023 | Myxochromide\_biosynthetic\_gene\_cluster | 26 | 63.8466622604 | 300 | 1e-80 |
| CAE53350.1 | non-ribosomal\_peptide\_synthetase | BGC0000440 | Teicoplanin\_biosynthetic\_gene\_cluster | 28 | 60.343688037 | 299 | 1.8e-80 |
| AEI58865.1 | peptide\_synthetase | BGC0000455 | Vancomycin\_biosynthetic\_gene\_cluster | 28 | 58.5591539987 | 299 | 2.3e-80 |
| CAG15009.1 | peptide\_synthetase,\_module\_1-2 | BGC0000441 | Teicoplanin\_biosynthetic\_gene\_cluster | 28 | 60.343688037 | 299 | 2.3e-80 |
| ALV86867.1 | Tlo21 | BGC0001406 | Telomycin\_biosynthetic\_gene\_cluster | 28 | 65.5651024455 | 299 | 3e-80 |
| CAD91211.1 | putative\_non-ribosomal\_peptide\_synthetase,\_module\_7 | BGC0000289 | A40926\_biosynthetic\_gene\_cluster | 28 | 64.24322538 | 299 | 3e-80 |
| CAJ34374.1 | NRPS\_protein | BGC0000445 | Thiocoraline\_biosynthetic\_gene\_cluster | 26 | 71.777924653 | 299 | 3e-80 |
| AGS77307.1 | NRPS\_modules\_1-2 | BGC0001178 | UK-68,597\_biosynthetic\_gene\_cluster | 28 | 63.5822868473 | 299 | 3e-80 |
| AAU39359.1 | lichenysin\_synthase\_LchAA | BGC0000381 | Lichenysin\_biosynthetic\_gene\_cluster | 26 | 64.4415069399 | 298 | 3.9e-80 |
| AHH53507.1 | non-ribosomal\_peptide\_synthetase | BGC0000439 | Taromycin\_biosynthetic\_gene\_cluster | 27 | 72.3727693325 | 298 | 5.1e-80 |
| ERG36783.1 | phenylalanine\_racemase | BGC0000299 | Alterochromides\_biosynthetic\_gene\_cluster | 26 | 70.7865168539 | 298 | 5.1e-80 |
| CAA11796.1 | PCZA363.5 | BGC0000322 | Chloroeremomycin\_biosynthetic\_gene\_cluster | 29 | 65.1685393258 | 298 | 5.1e-80 |
| AJF34463.1 | Txo1 | BGC0001207 | Teixobactin\_biosynthetic\_gene\_cluster | 25 | 91.8043621943 | 297 | 6.7e-80 |
| AGZ15458.1 | putative\_non-ribosomal\_peptide\_synthetase | BGC0001036 | Polyoxypeptin\_biosynthetic\_gene\_cluster | 27 | 63.6483807006 | 297 | 6.7e-80 |
| CAB53322.1 | putative\_peptide\_synthetase | BGC0000325 | Coelichelin\_biosynthetic\_gene\_cluster | 27 | 72.2405816259 | 297 | 8.8e-80 |
| AAX31559.1 | peptide\_synthetase\_3 | BGC0000336 | Daptomycin\_biosynthetic\_gene\_cluster | 26 | 79.9074686054 | 297 | 8.8e-80 |
| AAZ23076.1 | peptide\_synthetase | BGC0000291 | A54145\_biosynthetic\_gene\_cluster | 25 | 89.4910773298 | 296 | 1.5e-79 |
| CCJ67637.1 | TaaB | BGC0000447 | Tolaasin\_biosynthetic\_gene\_cluster | 26 | 78.6516853933 | 296 | 1.5e-79 |
| AID65222.1 | putative\_aspartate\_racemase | BGC0000335 | Cystomanamides\_biosynthetic\_gene\_cluster | 27 | 64.7719762062 | 296 | 1.9e-79 |
| CAG15012.1 | peptide\_synthetase,\_module\_7 | BGC0000441 | Teicoplanin\_biosynthetic\_gene\_cluster | 27 | 73.6946463979 | 295 | 3.3e-79 |
| BAH43869.1 | linear\_pentadecapeptide\_gramicidin\_synthetase\_LgrA | BGC0000367 | Gramicidin\_biosynthetic\_gene\_cluster | 25 | 76.9993390615 | 295 | 3.3e-79 |
| ABV79985.1 | ApnA | BGC0000301 | Anabaenopeptin\_biosynthetic\_gene\_cluster | 30 | 57.7660277594 | 295 | 4.3e-79 |
| AJF34464.1 | Txo2 | BGC0001207 | Teixobactin\_biosynthetic\_gene\_cluster | 29 | 66.9530733642 | 295 | 4.3e-79 |
| CAE53353.1 | non-ribosomal\_peptide\_synthetase | BGC0000440 | Teicoplanin\_biosynthetic\_gene\_cluster | 27 | 73.6946463979 | 295 | 4.3e-79 |
| AFH75329.1 | nonribosomal\_peptide\_synthetase | BGC0000398 | Orfamide\_biosynthetic\_gene\_cluster | 26 | 81.6920026438 | 295 | 4.3e-79 |
| CBD77749.1 | non-ribosomal\_peptide\_synthetase | BGC0000974 | Crocacin\_biosynthetic\_gene\_cluster | 26 | 87.6404494382 | 294 | 7.4e-79 |
| AIE77060.1 | peptide\_synthetase\_module\_7 | BGC0000418 | Ristocetin\_biosynthetic\_gene\_cluster | 27 | 77.3298083278 | 294 | 7.4e-79 |
| ACA09733.2 | fusaricidin\_synthetase | BGC0000358 | Fusaricidin\_biosynthetic\_gene\_cluster | 24 | 89.4249834765 | 294 | 9.7e-79 |
| AGS77310.1 | NRPS\_module\_7 | BGC0001178 | UK-68,597\_biosynthetic\_gene\_cluster | 27 | 74.2233972241 | 294 | 9.7e-79 |
| CAC48360.1 | peptide\_synthetase | BGC0000311 | Balhimycin\_biosynthetic\_gene\_cluster | 28 | 58.4269662921 | 292 | 2.2e-78 |
| AMK48228.1 | nonribosomal\_peptide\_synthetase | BGC0001351 | Cahuitamycins\_biosynthetic\_gene\_cluster | 28 | 64.6397884997 | 292 | 2.8e-78 |
| AJM89735.1 | PmxA | BGC0001192 | Colistin\_biosynthetic\_gene\_cluster | 32 | 40.7799074686 | 292 | 2.8e-78 |
| CBJ90082.1 | Non\_Ribosomal\_peptide\_synthetase\_(-succinylbenzoate--CoA\_ligase) | BGC0001132 | Xenotetrapeptide\_biosynthetic\_gene\_cluster | 25 | 89.5571711831 | 292 | 2.8e-78 |
| ADA69239.2 | trans-AT\_hybrid\_polyketide\_synthase-NRPS | BGC0001071 | Nosperin\_biosynthetic\_gene\_cluster | 27 | 64.9041639128 | 292 | 2.8e-78 |
| AAY37653.1 | Amino\_acid\_adenylation | BGC0000437 | Syringomycin\_biosynthetic\_gene\_cluster | 24 | 94.9768671514 | 292 | 2.8e-78 |
| CAD17793.1 | probable\_non\_ribosomal\_peptide\_synthetase\_protein | BGC0001363 | Ralsolamycin\_biosynthetic\_gene\_cluster | 26 | 72.1744877726 | 291 | 4.8e-78 |
| AIG79240.1 | Hypothetical\_protein | BGC0000419 | Ristomycin\_A\_biosynthetic\_gene\_cluster | 27 | 77.3298083278 | 291 | 4.8e-78 |
| CCJ67638.1 | TaaC | BGC0000447 | Tolaasin\_biosynthetic\_gene\_cluster | 24 | 94.9768671514 | 291 | 6.3e-78 |
| AGC45618.1 | non-ribosomal\_peptide\_synthetase | BGC0001394 | Phenalamide\_biosynthetic\_gene\_cluster | 30 | 48.5789821547 | 291 | 6.3e-78 |
| AJI44167.1 | long-chain-fatty-acid-CoA\_ligase | BGC0001193 | Thiolutin\_biosynthetic\_gene\_cluster | 29 | 60.0132187707 | 290 | 8.2e-78 |
| AHZ34233.1 | CifB | BGC0000323 | Cichofactin\_biosynthetic\_gene\_cluster | 24 | 99.1407799075 | 290 | 8.2e-78 |
| CBJ79915.1 | putative\_Phenylalanine\_racemase\_(ATP-hydrolyzing) | BGC0001133 | Taxlllaid\_biosynthetic\_gene\_cluster | 28 | 66.6226040978 | 290 | 1.1e-77 |
| AHZ34232.1 | CifA | BGC0000323 | Cichofactin\_biosynthetic\_gene\_cluster | 27 | 66.8208856576 | 290 | 1.4e-77 |
| AEW31019.1 | plipastatin\_synthetase | BGC0000407 | Plipastatin\_biosynthetic\_gene\_cluster | 24 | 89.5571711831 | 290 | 1.4e-77 |
| ACG60782.1 | NRPS(C/A/PCP/C/A/PCP) | BGC0001058 | Zorbamycin\_biosynthetic\_gene\_cluster | 26 | 65.0363516193 | 289 | 1.8e-77 |
| ABL74936.1 | TlmX | BGC0001048 | Tallysomycin\_biosynthetic\_gene\_cluster | 28 | 62.7891606081 | 289 | 1.8e-77 |
| CAJ77696.1 | MPS2\_protein | BGC0000363 | Glycopeptidolipid\_biosynthetic\_gene\_cluster | 27 | 62.1943159286 | 289 | 2.4e-77 |
| AHD05679.1 | putative\_non-ribosomal\_peptide\_ligase/\_polyketide\_synthase\_hybrid | BGC0000402 | Paenilarvins\_biosynthetic\_gene\_cluster | 27 | 65.5651024455 | 289 | 3.1e-77 |
| CCJ67639.1 | TaaD | BGC0000447 | Tolaasin\_biosynthetic\_gene\_cluster | 26 | 80.2379378718 | 289 | 3.1e-77 |
| AFH75321.1 | nonribosomal\_peptide\_synthetase | BGC0000425 | Sessilin\_biosynthetic\_gene\_cluster | 24 | 94.646397885 | 288 | 4.1e-77 |
| AAY37647.1 | Amino\_acid\_adenylation | BGC0000437 | Syringomycin\_biosynthetic\_gene\_cluster | 24 | 89.6232650364 | 288 | 5.3e-77 |
| CAD91220.1 | putative\_non-ribosomal\_peptide\_synthetase,\_modules\_1-2 | BGC0000289 | A40926\_biosynthetic\_gene\_cluster | 29 | 60.6080634501 | 288 | 5.3e-77 |
| ABS74207.1 | FenC | BGC0001095 | Fengycin\_biosynthetic\_gene\_cluster | 24 | 96.8935888962 | 288 | 5.3e-77 |
| AEW31021.1 | plipastatin\_synthetase | BGC0000407 | Plipastatin\_biosynthetic\_gene\_cluster | 25 | 74.4216787839 | 288 | 5.3e-77 |
| ctg1\_orf19 |  | BGC0001013 | Meridamycin\_biosynthetic\_gene\_cluster | 26 | 80.8327825512 | 287 | 6.9e-77 |
| AKC91849.1 | nonribosomal\_peptide\_synthetase | BGC0001414 | Griselimycin\_biosynthetic\_gene\_cluster | 25 | 79.8413747521 | 287 | 6.9e-77 |
| ABC87508.1 | NRPS\_for\_pipecolate\_incorporation | BGC0001011 | Meridamycin\_biosynthetic\_gene\_cluster | 26 | 80.8327825512 | 287 | 6.9e-77 |
| AAC06347.1 | bacitracin\_synthetase\_2 | BGC0000310 | Bacitracin\_biosynthetic\_gene\_cluster | 22 | 102.247191011 | 287 | 9.1e-77 |
| AEZ51516.1 | PmxA | BGC0001153 | Polymyxin\_biosynthetic\_gene\_cluster | 32 | 40.6477197621 | 287 | 9.1e-77 |
| ABI26078.1 | OciB | BGC0000331 | Cyanopeptin\_biosynthetic\_gene\_cluster | 26 | 71.7118307997 | 286 | 1.5e-76 |
| AJW76711.1 | DsaI | BGC0001196 | Desotamide\_biosynthetic\_gene\_cluster | 27 | 61.4672835426 | 286 | 2e-76 |
| AEU11006.1 | NpnB | BGC0001029 | Nostophycin\_biosynthetic\_gene\_cluster | 25 | 72.1083939194 | 286 | 2e-76 |
| AEI70245.1 | nonribosomal\_peptide\_synthetase\_NRPS | BGC0000401 | Paenibactin\_biosynthetic\_gene\_cluster | 24 | 103.43688037 | 285 | 4.5e-76 |
| ALV82356.1 | CDA\_peptide\_synthetase\_I | BGC0001370 | Lipopeptide\_8D1-1\_/\_lipopeptide\_8D1-2\_biosynthetic\_gene\_cluster | 27 | 64.24322538 | 285 | 4.5e-76 |
| AHD05618.1 | putative\_non-ribosomal\_peptide\_ligase\_domain\_protein | BGC0001033 | Paenilamicin\_biosynthetic\_gene\_cluster | 24 | 71.6457369465 | 285 | 4.5e-76 |
| AAF08795.1 | MycA | BGC0001103 | Mycosubtilin\_biosynthetic\_gene\_cluster | 27 | 68.3410442829 | 284 | 5.9e-76 |
| AHD05615.1 | putative\_non-ribosomal\_peptide\_ligase/\_polyketide\_synthase\_hybrid | BGC0001033 | Paenilamicin\_biosynthetic\_gene\_cluster | 23 | 94.9768671514 | 284 | 7.7e-76 |
| AFH75322.1 | nonribosomal\_peptide\_synthetase | BGC0000425 | Sessilin\_biosynthetic\_gene\_cluster | 25 | 87.0456047588 | 284 | 7.7e-76 |
| BAH22764.1 | nonribosomal\_peptide\_synthetase | BGC0001018 | Micropeptin\_biosynthetic\_gene\_cluster | 28 | 50.8261731659 | 283 | 1.3e-75 |
| AID65225.1 | nonribosomal\_peptide\_synthetase | BGC0000335 | Cystomanamides\_biosynthetic\_gene\_cluster | 25 | 77.131526768 | 282 | 2.9e-75 |
| AFJ23825.1 | WLIP\_synthetase\_B | BGC0000462\_c2 | WLIP\_biosynthetic\_gene\_cluster | 25 | 95.2412425644 | 282 | 3.8e-75 |
| AEP18656.1 | WAPS1 | BGC0000461 | WAP-8294A2\_(lotilibcin)\_biosynthetic\_gene\_cluster | 28 | 66.3582286847 | 281 | 5e-75 |
| ABW17376.1 | PsoB | BGC0000411 | Putisolvin\_biosynthetic\_gene\_cluster | 26 | 78.8499669531 | 281 | 5e-75 |
| ADD82940.1 | Bat2 | BGC0001099 | Kalimantacin\_/\_batumin\_biosynthetic\_gene\_cluster | 25 | 73.231989425 | 281 | 5e-75 |
| ABC35522.1 | thiotemplate\_mechanism\_natural\_product\_synthetase | BGC0000186 | Thailandamide\_biosynthetic\_gene\_cluster | 25 | 72.0423000661 | 281 | 6.5e-75 |
| KPN90369.1 | NunE | BGC0001416 | Nunapeptin\_/\_nunamycin\_biosynthetic\_gene\_cluster | 23 | 87.5082617317 | 280 | 1.1e-74 |
| CAA11794.1 | PCZA363.3 | BGC0000322 | Chloroeremomycin\_biosynthetic\_gene\_cluster | 27 | 58.3608724389 | 280 | 1.1e-74 |
| AAO62588.1 | peptide\_sythetase | BGC0001016 | Microcystin\_biosynthetic\_gene\_cluster | 24 | 72.4388631857 | 280 | 1.1e-74 |
| ABV79987.1 | ApnC | BGC0000301 | Anabaenopeptin\_biosynthetic\_gene\_cluster | 27 | 50.363516193 | 279 | 1.9e-74 |
| AAG02355.1 | peptide\_synthetase\_NRPS9-8 | BGC0000963 | Bleomycin\_biosynthetic\_gene\_cluster | 27 | 64.5076007931 | 279 | 1.9e-74 |
| ACY06285.1 | non-ribosomal\_peptide\_synthetase | BGC0001042 | Sanglifehrin\_A\_biosynthetic\_gene\_cluster | 27 | 64.5736946464 | 279 | 1.9e-74 |
| AAY91420.2 | non-ribosomal\_peptide\_synthetase\_OfaB | BGC0000399 | Orfamide\_biosynthetic\_gene\_cluster | 27 | 71.9762062128 | 279 | 2.5e-74 |
| AAZ55899.1 | amino\_acid\_adenylation | BGC0000359 | Fuscachelin\_biosynthetic\_gene\_cluster | 27 | 73.4963648381 | 279 | 2.5e-74 |
| AAZ23078.1 | peptide\_synthetase | BGC0000291 | A54145\_biosynthetic\_gene\_cluster | 26 | 65.2346331791 | 279 | 2.5e-74 |
| AEZ51520.1 | PmxE | BGC0001153 | Polymyxin\_biosynthetic\_gene\_cluster | 25 | 80.304031725 | 278 | 5.5e-74 |
| CAN89663.1 | putative\_non-ribosomal\_peptide\_synthetase | BGC0001070 | Kirromycin\_biosynthetic\_gene\_cluster | 27 | 64.970257766 | 277 | 7.2e-74 |
| ACZ55945.1 | non-ribosomal\_peptide\_synthetase | BGC0000302 | Anabaenopeptin\_biosynthetic\_gene\_cluster | 30 | 46.2656972902 | 277 | 7.2e-74 |
| CAG29032.1 | nonribosomal\_peptide\_synthetase\_(modules\_3\_to\_6) | BGC0001023 | Myxochromide\_biosynthetic\_gene\_cluster | 26 | 78.7838730998 | 277 | 9.4e-74 |
| CAJ18237.2 | non-ribosomal\_peptide\_synthetase\_B | BGC0000354 | Friulimicin\_biosynthetic\_gene\_cluster | 26 | 82.8155981494 | 277 | 1.2e-73 |
| AGM16414.1 | paenibacterin\_synthetase\_C | BGC0000400 | Paenibacterin\_biosynthetic\_gene\_cluster | 26 | 64.7058823529 | 276 | 1.6e-73 |
| ALK27916.1 | non-ribosomal\_peptide\_synthase | BGC0001233 | Feglymycin\_biosynthetic\_gene\_cluster | 30 | 45.8691341705 | 276 | 1.6e-73 |
| AAX31558.1 | peptide\_synthetase\_2 | BGC0000336 | Daptomycin\_biosynthetic\_gene\_cluster | 26 | 66.2921348315 | 276 | 2.1e-73 |
| ABV56588.1 | KtzH | BGC0000378 | Kutznerides\_biosynthetic\_gene\_cluster | 27 | 61.0046265697 | 276 | 2.1e-73 |
| ANQ43347.1 | non-ribosomal\_peptide\_synthetase | BGC0001347 | Poaeamide\_B\_biosynthetic\_gene\_cluster | 27 | 71.6457369465 | 275 | 4.7e-73 |
| ACA97580.1 | PmxE | BGC0000408 | Polymyxin\_biosynthetic\_gene\_cluster | 24 | 80.1057501652 | 274 | 6.1e-73 |
| AEG64696.1 | LpmB | BGC0000379 | Laspartomycin\_biosynthetic\_gene\_cluster | 26 | 65.3007270324 | 274 | 6.1e-73 |
| WP\_010369430.1 | Non-ribosomal\_peptide\_synthase | BGC0000314 | Bromoalterochromides\_biosynthetic\_gene\_cluster | 28 | 56.9068076669 | 274 | 7.9e-73 |
| CCJ67648.1 | JagD | BGC0001127 | Jagaricin\_biosynthetic\_gene\_cluster | 27 | 62.8552544613 | 274 | 7.9e-73 |
| AAK57184.1 | MxaA | BGC0001022 | Myxalamid\_biosynthetic\_gene\_cluster | 31 | 46.3978849967 | 273 | 1.4e-72 |
| AHD05677.1 | nonribosomal\_peptide\_ligase\_subunit | BGC0000402 | Paenilarvins\_biosynthetic\_gene\_cluster | 27 | 59.4844679445 | 273 | 1.4e-72 |
| AIG79243.1 | Hypothetical\_protein | BGC0000419 | Ristomycin\_A\_biosynthetic\_gene\_cluster | 27 | 59.8810310641 | 272 | 3e-72 |
| BAH22762.1 | nonribosomal\_peptide\_synthetase | BGC0001018 | Micropeptin\_biosynthetic\_gene\_cluster | 25 | 72.9676140119 | 271 | 5.1e-72 |
| CAB38518.1 | CDA\_peptide\_synthetase\_I | BGC0000315 | Calcium-dependent\_antibiotic\_biosynthetic\_gene\_cluster | 27 | 65.0363516193 | 271 | 5.1e-72 |
| AMK48226.1 | nonribosomal\_peptide\_synthetase | BGC0001351 | Cahuitamycins\_biosynthetic\_gene\_cluster | 27 | 65.0363516193 | 271 | 5.1e-72 |
| CAN89656.1 | putative\_non-ribosomal\_peptide\_synthetase | BGC0001070 | Kirromycin\_biosynthetic\_gene\_cluster | 26 | 74.0912095175 | 271 | 5.1e-72 |
| AJM89738.1 | PmxE | BGC0001192 | Colistin\_biosynthetic\_gene\_cluster | 24 | 80.039656312 | 271 | 6.7e-72 |
| AEC14347.1 | nonribosomal\_peptide\_synthetase | BGC0000377 | Koranimine\_biosynthetic\_gene\_cluster | 26 | 63.8466622604 | 270 | 8.8e-72 |
| ABA70582.1 | alpha-aminoadypil-cysteinyl-valine\_synthetase | BGC0000404 | Penicillin\_biosynthetic\_gene\_cluster | 25 | 79.8413747521 | 270 | 8.8e-72 |
| KFH48607.1 | N-like\_protein | BGC0000317 | Cephalosporin\_biosynthetic\_gene\_cluster | 25 | 91.5399867812 | 270 | 8.8e-72 |
| AGN74886.1 | nonribosomal\_peptide\_synthetase | BGC0000459 | Griseoviridin\_/\_viridogrisein\_biosynthetic\_gene\_cluster | 28 | 64.3754130866 | 270 | 8.8e-72 |
| ANI24100.1 | nonribosomal\_peptide\_synthetase | BGC0001235 | Antalid\_biosynthetic\_gene\_cluster | 26 | 64.1110376735 | 270 | 1.1e-71 |
| ALV86866.1 | Tlo20 | BGC0001406 | Telomycin\_biosynthetic\_gene\_cluster | 27 | 66.7547918044 | 270 | 1.1e-71 |
| AAX31557.1 | peptide\_synthetase\_1 | BGC0000336 | Daptomycin\_biosynthetic\_gene\_cluster | 29 | 57.0389953734 | 270 | 1.1e-71 |
| KPN93065.1 | NunD | BGC0001416 | Nunapeptin\_/\_nunamycin\_biosynthetic\_gene\_cluster | 26 | 63.9127561137 | 270 | 1.5e-71 |
| ABR12615.1 | ACV\_synthetase | BGC0000405 | Penicillin\_biosynthetic\_gene\_cluster | 25 | 79.8413747521 | 269 | 2e-71 |
| AAY93355.1 | non-ribosomal\_peptide\_synthetase\_PvdJ | BGC0000413 | Pyoverdine\_biosynthetic\_gene\_cluster | 26 | 64.970257766 | 269 | 3.3e-71 |
| AHD05627.1 | putative\_non-ribosomal\_peptide\_ligase\_domain\_protein | BGC0001033 | Paenilamicin\_biosynthetic\_gene\_cluster | 29 | 48.1163251818 | 268 | 5.7e-71 |
| BAC67535.1 | arthrofactin\_synthetase\_B | BGC0000305 | Arthrofactin\_biosynthetic\_gene\_cluster | 24 | 79.1143423662 | 267 | 7.4e-71 |
| AEH59100.1 | amino\_acid\_adenylation\_domain-containing\_protein/NRPS | BGC0000385 | Lysobactin\_biosynthetic\_gene\_cluster | 26 | 65.4329147389 | 267 | 9.7e-71 |
| EFL06865.1 | hypothetical\_protein | BGC0000300 | Amychelin\_biosynthetic\_gene\_cluster | 25 | 71.9762062128 | 267 | 1.3e-70 |
| AFJ23819.1 | WLIP\_synthetase\_A | BGC0000462 | WLIP\_biosynthetic\_gene\_cluster | 27 | 67.0191672174 | 267 | 1.3e-70 |
| AGZ15459.1 | putative\_non-ribosomal\_peptide\_synthetase | BGC0001036 | Polyoxypeptin\_biosynthetic\_gene\_cluster | 27 | 64.5076007931 | 267 | 1.3e-70 |
| CCJ67640.1 | TaaE | BGC0000447 | Tolaasin\_biosynthetic\_gene\_cluster | 27 | 57.17118308 | 267 | 1.3e-70 |
| BAP05596.1 | CalH | BGC0000967 | Calyculin\_biosynthetic\_gene\_cluster | 26 | 64.8380700595 | 266 | 1.7e-70 |
| CAE02633.1 | surfactin\_synthetase\_C | BGC0000433 | Surfactin\_biosynthetic\_gene\_cluster | 23 | 73.0337078652 | 265 | 2.8e-70 |
| AAO56328.1 | non-ribosomal\_peptide\_synthetase\_SyfA | BGC0000435 | Syringafactin\_biosynthetic\_gene\_cluster | 27 | 66.3582286847 | 265 | 3.7e-70 |
| AGE26255.1 | putative\_non-ribosomal\_peptide\_synthetase | BGC0001208 | Poaeamide\_biosynthetic\_gene\_cluster | 26 | 72.1744877726 | 265 | 3.7e-70 |
| AHI59110.1 | locillomycin\_synthase\_C | BGC0001005 | Locillomycin\_biosynthetic\_gene\_cluster | 25 | 63.9127561137 | 265 | 3.7e-70 |
| ALV82384.1 | CDA\_peptide\_synthetase\_II | BGC0001370 | Lipopeptide\_8D1-1\_/\_lipopeptide\_8D1-2\_biosynthetic\_gene\_cluster | 31 | 48.2485128883 | 265 | 4.8e-70 |
| ABF87031.1 | non-ribosomal\_peptide\_synthetase/polyketide\_synthase | BGC0000393 | Myxoprincomide\_biosynthetic\_gene\_cluster | 27 | 65.9616655651 | 264 | 6.3e-70 |
| ADZ24999.1 | non-ribosomal\_peptide\_synthase | BGC0000380 | Leupyrrin\_biosynthetic\_gene\_cluster | 27 | 64.7719762062 | 264 | 8.2e-70 |
| AEH59099.1 | amino\_acid\_adenylation\_domain-containing\_protein/NRPS | BGC0000385 | Lysobactin\_biosynthetic\_gene\_cluster | 26 | 66.6226040978 | 264 | 1.1e-69 |
| ABW17375.1 | PsoA | BGC0000411 | Putisolvin\_biosynthetic\_gene\_cluster | 27 | 64.1110376735 | 264 | 1.1e-69 |
| ABS74181.1 | BmyA | BGC0001090 | Bacillomycin\_biosynthetic\_gene\_cluster | 30 | 43.8863185724 | 264 | 1.1e-69 |
| AGM14933.1 | xantholysin\_synthetase\_B | BGC0000463 | Xantholysin\_biosynthetic\_gene\_cluster | 25 | 70.9847984137 | 263 | 1.4e-69 |
| AIG79242.1 | Hypothetical\_protein | BGC0000419 | Ristomycin\_A\_biosynthetic\_gene\_cluster | 26 | 65.1024454726 | 263 | 1.8e-69 |
| AAG02359.1 | peptide\_synthetase\_NRPS5-4-3 | BGC0000963 | Bleomycin\_biosynthetic\_gene\_cluster | 25 | 89.4249834765 | 262 | 2.4e-69 |
| ANQ43348.1 | non-ribosomal\_peptide\_synthetase | BGC0001347\_c2 | Poaeamide\_B\_biosynthetic\_gene\_cluster | 24 | 88.1692002644 | 262 | 2.4e-69 |
| AFD30953.1 | CrmB | BGC0000966 | Caerulomycin\_A\_biosynthetic\_gene\_cluster | 25 | 75.0826173166 | 262 | 2.4e-69 |
| CAG23960.2 | hybrid\_NRPS/PKS | BGC0001089 | Bacillaene\_biosynthetic\_gene\_cluster | 26 | 72.4388631857 | 262 | 2.4e-69 |
| BAC67534.2 | arthrofactin\_synthetase\_A | BGC0000305 | Arthrofactin\_biosynthetic\_gene\_cluster | 26 | 65.6311962987 | 262 | 2.4e-69 |
| WP\_051700111.1 | non-ribosomal\_peptide\_synthetase | BGC0001368 | Tambromycin\_biosynthetic\_gene\_cluster | 26 | 62.7891606081 | 262 | 3.1e-69 |
| ABL74940.1 | TlmVI | BGC0001048 | Tallysomycin\_biosynthetic\_gene\_cluster | 25 | 81.6259087905 | 262 | 4.1e-69 |
| BAB69698.1 | iturin\_A\_synthetase\_A | BGC0001098 | Iturin\_biosynthetic\_gene\_cluster | 30 | 43.8863185724 | 262 | 4.1e-69 |
| AFJ14794.1 | PlpE | BGC0000403 | Pelgipeptin\_biosynthetic\_gene\_cluster | 29 | 48.4467944481 | 262 | 4.1e-69 |
| ALV82388.1 | CDA\_peptide\_synthetase\_III | BGC0001370 | Lipopeptide\_8D1-1\_/\_lipopeptide\_8D1-2\_biosynthetic\_gene\_cluster | 28 | 65.7633840053 | 262 | 4.1e-69 |
| CAB15186.3 | siderophore\_2,3-dihydroxybenzoate-glycine-threonine\_trimeric\_ester\_bacillibactin\_synthetase | BGC0000309 | Bacillibactin\_biosynthetic\_gene\_cluster | 27 | 66.8208856576 | 262 | 4.1e-69 |
| CCM44337.1 | putative\_nonribosomal\_peptide\_synthetase | BGC0001056 | Zeamine\_biosynthetic\_gene\_cluster | 24 | 83.6087243886 | 262 | 4.1e-69 |
| BAE98155.1 | putative\_non-ribosomal\_peptide\_synthetase | BGC0000339 | Echinomycin\_biosynthetic\_gene\_cluster | 27 | 68.8697951091 | 261 | 7e-69 |
| AET13879.1 | epichloenin\_A\_synthetase | BGC0001251 | Epichloenin\_A\_biosynthetic\_gene\_cluster | 24 | 82.4190350297 | 260 | 1.2e-68 |
| AAT01807.1 | non-ribosomal\_peptide\_synthetase | BGC0000365 | Glycopeptidolipid\_biosynthetic\_gene\_cluster | 32 | 43.0270984798 | 260 | 1.2e-68 |
| AAO56329.1 | non-ribosomal\_peptide\_synthetase\_SyfB | BGC0000435 | Syringafactin\_biosynthetic\_gene\_cluster | 27 | 62.5908790482 | 260 | 1.5e-68 |
| AFH75330.1 | nonribosomal\_peptide\_synthetase | BGC0000398 | Orfamide\_biosynthetic\_gene\_cluster | 25 | 73.8268341044 | 259 | 2e-68 |
| CAJ34381.1 | NRPS\_protein | BGC0000445 | Thiocoraline\_biosynthetic\_gene\_cluster | 26 | 65.3007270324 | 259 | 2.6e-68 |
| CAJ77716.1 | Mps2\_protein | BGC0000364 | Glycopeptidolipid\_biosynthetic\_gene\_cluster | 31 | 42.6966292135 | 259 | 2.6e-68 |
| AAT09804.1 | NocA | BGC0000395 | Nocardicin\_A\_biosynthetic\_gene\_cluster | 28 | 63.0535360212 | 259 | 2.6e-68 |
| ACU71638.1 | amino\_acid\_adenylation\_domain\_protein | BGC0001154 | Cacibiocin\_biosynthetic\_gene\_cluster | 31 | 39.9867812293 | 259 | 3.5e-68 |
| CBD77746.1 | non-ribosomal\_peptide\_synthetase/polyketide\_synthase | BGC0000974 | Crocacin\_biosynthetic\_gene\_cluster | 26 | 63.978849967 | 258 | 4.5e-68 |
| ABX37383.1 | amino\_acid\_adenylation\_domain\_protein | BGC0000984 | Delftibactin\_biosynthetic\_gene\_cluster | 25 | 72.7032385988 | 258 | 4.5e-68 |
| CBJ79916.1 | putative\_Ornithine\_racemase | BGC0001133 | Taxlllaid\_biosynthetic\_gene\_cluster | 23 | 91.6721744878 | 258 | 4.5e-68 |
| AFR69334.1 | nonribosomal\_peptide\_synthetase\_SpiDE1 | BGC0001045 | Spiruchostatin\_biosynthetic\_gene\_cluster | 26 | 63.0535360212 | 258 | 5.9e-68 |
| ACM79810.1 | ZmaO | BGC0001059 | Zwittermycin\_A\_biosynthetic\_gene\_cluster | 24 | 64.8380700595 | 257 | 1.3e-67 |
| CAB38517.1 | CDA\_peptide\_synthetase\_II | BGC0000315 | Calcium-dependent\_antibiotic\_biosynthetic\_gene\_cluster | 30 | 47.5875743556 | 257 | 1.3e-67 |
| AKC91856.1 | nonribosomal\_peptide\_synthetase | BGC0001414 | Griselimycin\_biosynthetic\_gene\_cluster | 27 | 65.1024454726 | 256 | 2.2e-67 |
| sln7 | nonribosomal\_peptide\_synthetase | BGC0001230 | Salinamides\_biosynthetic\_gene\_cluster | 27 | 68.0105750165 | 255 | 2.9e-67 |
| AGM14934.1 | xantholysin\_synthetase\_C | BGC0000463 | Xantholysin\_biosynthetic\_gene\_cluster | 24 | 82.9477858559 | 254 | 8.5e-67 |
| AAN85512.1 | nonribosomal\_peptide\_synthetase | BGC0001101 | Leinamycin\_biosynthetic\_gene\_cluster | 26 | 65.4990085922 | 253 | 1.1e-66 |
| CAJ88610.1 | putative\_non-ribosomal\_peptide\_synthetase | BGC0000327 | Congocidine\_biosynthetic\_gene\_cluster | 27 | 66.2260409782 | 253 | 1.1e-66 |
| ABP57748.1 | DepD | BGC0000993 | FK228\_biosynthetic\_gene\_cluster | 25 | 71.7118307997 | 253 | 1.9e-66 |
| ABS74208.1 | FenB | BGC0001095 | Fengycin\_biosynthetic\_gene\_cluster | 24 | 78.2551222736 | 253 | 1.9e-66 |
| CAD55498.1 | CDA\_peptide\_synthetase\_III | BGC0000315 | Calcium-dependent\_antibiotic\_biosynthetic\_gene\_cluster | 28 | 65.4329147389 | 252 | 2.5e-66 |
| ABS74209.1 | FenA | BGC0001095 | Fengycin\_biosynthetic\_gene\_cluster | 23 | 83.5426305354 | 252 | 2.5e-66 |
| CAF05648.1 | TubC\_protein | BGC0001053 | Tubulysin\_biosynthetic\_gene\_cluster | 26 | 65.2346331791 | 252 | 2.5e-66 |
| AAY32966.1 | DszC | BGC0001093 | Disorazole\_biosynthetic\_gene\_cluster | 32 | 42.4983476537 | 252 | 3.2e-66 |
| sln6 | nonribosomal\_peptide\_synthetase | BGC0001230 | Salinamides\_biosynthetic\_gene\_cluster | 27 | 64.7058823529 | 252 | 3.2e-66 |
| ABW00331.1 | amino\_acid\_adenylation\_domain | BGC0000333 | Cyclomarin\_biosynthetic\_gene\_cluster | 25 | 62.524785195 | 252 | 3.2e-66 |
| sln9 | nonribosomal\_peptide\_synthetase | BGC0001230 | Salinamides\_biosynthetic\_gene\_cluster | 25 | 72.1744877726 | 252 | 3.2e-66 |
| AAY91419.3 | non-ribosomal\_peptide\_synthetase\_OfaA | BGC0000399 | Orfamide\_biosynthetic\_gene\_cluster | 25 | 71.8440185063 | 251 | 7.2e-66 |
| AIE77058.1 | peptide\_synthetase\_module\_3 | BGC0000418 | Ristocetin\_biosynthetic\_gene\_cluster | 26 | 64.7058823529 | 250 | 9.4e-66 |
| AGN74885.1 | nonribosomal\_peptide\_synthetase | BGC0000459 | Griseoviridin\_/\_viridogrisein\_biosynthetic\_gene\_cluster | 25 | 78.6516853933 | 250 | 1.2e-65 |
| sln8 | nonribosomal\_peptide\_synthetase | BGC0001230 | Salinamides\_biosynthetic\_gene\_cluster | 26 | 64.6397884997 | 250 | 1.6e-65 |
| AAS92545.1 | SirP | BGC0001044 | Sirodesmin\_biosynthetic\_gene\_cluster | 26 | 50.7600793126 | 250 | 1.6e-65 |
| CCE28989.1 | non-ribosomal\_peptide\_synthetase | BGC0001365 | Clapurines\_biosynthetic\_gene\_cluster | 24 | 71.5135492399 | 250 | 1.6e-65 |
| ABV56585.1 | KtzE | BGC0000378 | Kutznerides\_biosynthetic\_gene\_cluster | 31 | 42.3000660939 | 247 | 8e-65 |
| AIE77076.1 | peptide\_synthetase | BGC0000418 | Ristocetin\_biosynthetic\_gene\_cluster | 33 | 39.9206873761 | 247 | 1e-64 |
| CAM02313.1 | putative\_non-ribosomal\_peptide\_synthetase | BGC0000349 | Erythrochelin\_biosynthetic\_gene\_cluster | 26 | 61.9960343688 | 247 | 1.4e-64 |
| ABI22131.1 | putative\_non-ribosomal\_peptide\_synthetase | BGC0000422 | Saframycin\_A\_biosynthetic\_gene\_cluster | 29 | 47.8519497687 | 247 | 1.4e-64 |
| AIC32693.1 | FR9DEF | BGC0001113 | Spliceostatin\_/\_FR901464\_biosynthetic\_gene\_cluster | 26 | 76.1401189689 | 245 | 3e-64 |
| ADH01485.1 | putative\_mixed\_polyketide\_synthase/non-ribosomal\_peptide\_synthetase | BGC0001096 | FR901464\_biosynthetic\_gene\_cluster | 26 | 76.1401189689 | 245 | 3e-64 |
| ABX37385.1 | amino\_acid\_adenylation\_domain\_protein | BGC0000984 | Delftibactin\_biosynthetic\_gene\_cluster | 28 | 55.717118308 | 245 | 3e-64 |
| CAJ45639.1 | vanchrobactin\_non\_ribosomal\_peptide\_synthetase | BGC0000454 | Vanchrobactin\_biosynthetic\_gene\_cluster | 25 | 63.7805684071 | 245 | 5.2e-64 |
| CAE14173.1 |  | BGC0000383 | Luminmycin\_biosynthetic\_gene\_cluster | 24 | 73.4302709848 | 245 | 5.2e-64 |
| AFJ14793.1 | PlpD | BGC0000403 | Pelgipeptin\_biosynthetic\_gene\_cluster | 25 | 67.151354924 | 245 | 5.2e-64 |
| AID65224.1 | nonribosomal\_peptide\_synthetase | BGC0000335 | Cystomanamides\_biosynthetic\_gene\_cluster | 23 | 76.6027759418 | 245 | 5.2e-64 |
| AET13875.1 | epichloenin\_A\_synthetase | BGC0001250 | Epichloenin\_A\_biosynthetic\_gene\_cluster | 26 | 66.0938532716 | 245 | 5.2e-64 |
| AGN11881.1 | TstDEF | BGC0001114 | Thailanstatin\_biosynthetic\_gene\_cluster | 27 | 70.1255783212 | 244 | 8.8e-64 |
| AEI58879.1 | peptide\_synthetase | BGC0000455 | Vancomycin\_biosynthetic\_gene\_cluster | 32 | 39.9867812293 | 242 | 3.3e-63 |
| BAD55612.1 | non-ribosomal\_peptide\_synthetase | BGC0001027 | Nocobactin\_NA\_biosynthetic\_gene\_cluster | 27 | 64.4415069399 | 241 | 7.4e-63 |
| ABP73646.1 | SalB | BGC0000145 | Salinosporamide\_A\_biosynthetic\_gene\_cluster | 29 | 41.3086582948 | 241 | 7.4e-63 |
| ABP53497.1 | amino\_acid\_adenylation\_domain | BGC0001041 | Salinosporamide\_biosynthetic\_gene\_cluster | 29 | 41.3086582948 | 241 | 7.4e-63 |
| EFE73308.1 | non-ribosomal\_peptide\_synthetase | BGC0000431 | Stenothricin\_biosynthetic\_gene\_cluster | 27 | 46.1996034369 | 239 | 2.2e-62 |
| AEF33078.1 | dimodular\_nonribosomal\_peptide\_synthetase | BGC0001039 | Pyridomycin\_biosynthetic\_gene\_cluster | 25 | 66.9530733642 | 239 | 2.2e-62 |
| CAM56771.1 |  | BGC0000354 | Friulimicin\_biosynthetic\_gene\_cluster | 26 | 72.7032385988 | 238 | 3.7e-62 |
| AHB82056.1 | non\_ribosomal\_peptide\_synthetase | BGC0001019 | Microsclerodermins\_biosynthetic\_gene\_cluster | 26 | 65.1685393258 | 238 | 4.8e-62 |
| CAJ76290.1 | putative\_non-ribosomal\_peptide\_synthase | BGC0000972 | Colibactin\_biosynthetic\_gene\_cluster | 25 | 73.8929279577 | 238 | 6.3e-62 |
| WP\_010369425.1 | ornithine\_racemase | BGC0000314 | Bromoalterochromides\_biosynthetic\_gene\_cluster | 30 | 42.4322538004 | 238 | 6.3e-62 |
| ERG36782.1 | Ornithine\_racemase | BGC0000299 | Alterochromides\_biosynthetic\_gene\_cluster | 30 | 42.4322538004 | 238 | 6.3e-62 |
| AKP45399.1 | CysK | BGC0001413 | Cystobactamide\_biosynthetic\_gene\_cluster | 25 | 65.4329147389 | 238 | 6.3e-62 |
| CAJ34382.1 | NRPS\_protein | BGC0000445 | Thiocoraline\_biosynthetic\_gene\_cluster | 24 | 71.9762062128 | 237 | 8.2e-62 |
| AAD44234.1 | PstB | BGC0000362 | Glycopeptidolipid\_biosynthetic\_gene\_cluster | 31 | 40.4494382022 | 237 | 1.1e-61 |
| AHB38497.1 | EpxD | BGC0000346 | Epoxomicin\_biosynthetic\_gene\_cluster | 25 | 66.0277594184 | 237 | 1.4e-61 |
| CAC11137.1 | NikP1\_protein | BGC0000876 | Nikkomycin\_biosynthetic\_gene\_cluster | 29 | 42.3661599471 | 237 | 1.4e-61 |
| AGC09528.1 | NRPS | BGC0001183 | Lobophorin\_biosynthetic\_gene\_cluster | 27 | 58.8235294118 | 236 | 2.4e-61 |
| AHH53508.1 | non-ribosomal\_peptide\_synthetase | BGC0000439 | Taromycin\_biosynthetic\_gene\_cluster | 27 | 56.7746199603 | 235 | 3.1e-61 |
| ABL86391.1 | hybrid\_polyketide\_synthase\_and\_nonribosomal\_peptide\_synthetase | BGC0000999 | Heat-stable\_antifungal\_factor\_biosynthetic\_gene\_cluster | 25 | 67.0191672174 | 235 | 4.1e-61 |
| ALD82525.1 | non-ribosomal\_peptide\_synthase | BGC0001212 | Nannocystin\_biosynthetic\_gene\_cluster | 27 | 50.8261731659 | 235 | 5.3e-61 |
| ALG65319.1 | Cal17 | BGC0001297 | WS9326\_biosynthetic\_gene\_cluster | 25 | 72.8354263054 | 234 | 7e-61 |
| CCM44336.1 | putative\_nonribosomal\_peptide\_synthetase | BGC0001056 | Zeamine\_biosynthetic\_gene\_cluster | 25 | 57.5677461996 | 234 | 9.1e-61 |
| CAD91221.1 | putative\_non-ribosomal\_peptide\_synthetase,\_module\_3 | BGC0000289 | A40926\_biosynthetic\_gene\_cluster | 28 | 52.2802379379 | 234 | 9.1e-61 |
| CCJ67647.1 | JagC | BGC0001127 | Jagaricin\_biosynthetic\_gene\_cluster | 24 | 70.7865168539 | 234 | 9.1e-61 |
| ADH04679.1 | non-ribosomal\_peptide\_synthetase | BGC0001344 | Tubulysin\_biosynthetic\_gene\_cluster | 25 | 62.1282220753 | 233 | 1.6e-60 |
| AAG02349.1 | peptide\_synthetase\_NRPS11-10 | BGC0000963 | Bleomycin\_biosynthetic\_gene\_cluster | 27 | 66.3582286847 | 232 | 2.6e-60 |
| AHD05617.1 | putative\_non-ribosomal\_peptide\_ligase\_domain\_protein | BGC0001033 | Paenilamicin\_biosynthetic\_gene\_cluster | 26 | 50.0991407799 | 232 | 2.6e-60 |
| ABD65956.1 | nonribosomal\_peptide\_synthetase | BGC0000341 | Enduracidin\_biosynthetic\_gene\_cluster | 29 | 44.5472571051 | 232 | 3.5e-60 |
| ABL74937.1 | TlmIX | BGC0001048 | Tallysomycin\_biosynthetic\_gene\_cluster | 26 | 64.1110376735 | 231 | 5.9e-60 |
| CBW54672.1 | non\_ribosomal\_peptide\_synthetase | BGC0000971 | Cinnabaramide\_biosynthetic\_gene\_cluster | 30 | 40.9120951751 | 231 | 5.9e-60 |
| BAJ19066.1 | L-lysine\_activating\_non-ribosomal\_peptide\_synthetase | BGC0000288 | A-503083\_biosynthetic\_gene\_cluster | 25 | 63.4500991408 | 230 | 1e-59 |
| ACG60773.1 | NRPS(C/A/PCP) | BGC0001058 | Zorbamycin\_biosynthetic\_gene\_cluster | 25 | 67.6140118969 | 230 | 1.3e-59 |
| CAC48369.1 | peptide\_synthetase | BGC0000311 | Balhimycin\_biosynthetic\_gene\_cluster | 37 | 27.6933245208 | 230 | 1.7e-59 |
| AHZ20784.1 | non-ribosomal\_peptide\_synthase | BGC0000369 | Hassallidins\_biosynthetic\_gene\_cluster | 27 | 52.2141440846 | 230 | 1.7e-59 |
| BAI63283.1 | putative\_non-ribosomal\_peptide\_synthetase | BGC0000434 | SW-163\_biosynthetic\_gene\_cluster | 30 | 40.2511566424 | 229 | 2.2e-59 |
| BAI23334.1 | putative\_non-ribosomal\_peptide\_synthetase | BGC0000949 | A-500359s\_biosynthetic\_gene\_cluster | 28 | 56.2458691342 | 229 | 2.9e-59 |
| ADN26248.1 | peptide\_synthetase | BGC0000951 | Pacidamycin\_biosynthetic\_gene\_cluster | 29 | 45.736946464 | 228 | 3.8e-59 |
| AGC65516.1 | TtbA | BGC0001050 | Thalassospiramide\_biosynthetic\_gene\_cluster | 25 | 62.6569729015 | 228 | 3.8e-59 |
| AEG64689.1 | LpmA | BGC0000379 | Laspartomycin\_biosynthetic\_gene\_cluster | 29 | 41.7713152677 | 228 | 5e-59 |
| AJM89734.1 | PmxB | BGC0001192 | Colistin\_biosynthetic\_gene\_cluster | 28 | 45.736946464 | 228 | 5e-59 |
| CBZ42143.1 | putative\_non-ribosomal\_peptide\_synthetase | BGC0001117 | Himastatin\_biosynthetic\_gene\_cluster | 31 | 41.837409121 | 228 | 6.5e-59 |
| AKP45395.1 | CysG | BGC0001413 | Cystobactamide\_biosynthetic\_gene\_cluster | 26 | 64.3093192333 | 228 | 6.5e-59 |
| AAC32047.1 | FxbB | BGC0000351 | Exochelin\_biosynthetic\_gene\_cluster | 30 | 43.8863185724 | 227 | 8.5e-59 |
| CCP45167.1 | Peptide\_synthetase\_MbtF\_(peptide\_synthase) | BGC0001021 | Mycobactin\_biosynthetic\_gene\_cluster | 26 | 68.0766688698 | 227 | 1.5e-58 |
| AGZ15460.1 | putative\_non-ribosomal\_peptide\_synthetase | BGC0001036 | Polyoxypeptin\_biosynthetic\_gene\_cluster | 27 | 52.2141440846 | 226 | 1.9e-58 |
| BAE56606.1 |  | BGC0001123 | Aspirochlorine\_biosynthetic\_gene\_cluster | 27 | 56.9068076669 | 225 | 3.2e-58 |
| AFP73394.1 | FusA | BGC0001268 | Fusarin\_biosynthetic\_gene\_cluster | 25 | 64.3093192333 | 225 | 4.2e-58 |
| ACN39015.1 | putative\_nonribosomal\_peptide\_synthetase\_TomB | BGC0000448 | Tomaymycin\_biosynthetic\_gene\_cluster | 24 | 69.2663582287 | 225 | 4.2e-58 |
| AEZ51517.1 | PmxB | BGC0001153 | Polymyxin\_biosynthetic\_gene\_cluster | 30 | 39.5902181097 | 225 | 4.2e-58 |
| CAK15814.1 | putative\_non-ribosomal\_peptide\_synthetase,\_terminal\_component | BGC0000344 | Entolysin\_biosynthetic\_gene\_cluster | 25 | 62.9874421679 | 225 | 5.5e-58 |
| ANQ43349.1 | non-ribosomal\_peptide\_synthetase | BGC0001347\_c2 | Poaeamide\_B\_biosynthetic\_gene\_cluster | 25 | 72.0423000661 | 224 | 9.4e-58 |
| ACA97577.1 | PmxB | BGC0000408 | Polymyxin\_biosynthetic\_gene\_cluster | 30 | 39.5902181097 | 224 | 9.4e-58 |
| CCA89328.1 | mixed\_trans-AT\_type\_I\_polyketide\_synthase/nonribosomal\_peptide\_synthetase | BGC0001111 | Rhizopodins\_biosynthetic\_gene\_cluster | 29 | 45.736946464 | 224 | 9.4e-58 |
| AGE25480.1 | amino\_acid\_adenylation\_protein | BGC0001208\_c2 | Poaeamide\_biosynthetic\_gene\_cluster | 25 | 71.3813615334 | 223 | 1.6e-57 |
| CCJ67645.1 | JagA | BGC0001127 | Jagaricin\_biosynthetic\_gene\_cluster | 28 | 55.8493060145 | 223 | 2.1e-57 |
| ABC94347.1 | vicibactin\_biosynthesis\_non-ribosomal\_peptide\_synthase\_protein | BGC0000457 | Vicibactin\_biosynthetic\_gene\_cluster | 28 | 47.455386649 | 223 | 2.1e-57 |
| AHH25592.1 | NRPS | BGC0000957 | Ansatrienin\_(mycotrienin)\_biosynthetic\_gene\_cluster | 32 | 42.8949107733 | 222 | 3.6e-57 |
| ABS90470.1 | NRPS/PKS | BGC0001106 | Oxazolomycin\_biosynthetic\_gene\_cluster | 26 | 56.5763384005 | 221 | 8e-57 |
| AAY89051.1 | hybrid\_nonribosomal\_peptide\_synthetase/polyketide\_synthase | BGC0001069 | Chivosazole\_biosynthetic\_gene\_cluster | 29 | 44.4150693985 | 221 | 8e-57 |
| ABH06368.2 | MassB | BGC0000389 | Massetolide\_A\_biosynthetic\_gene\_cluster | 27 | 50.0991407799 | 220 | 1.4e-56 |
| CAJ96468.1 | Non-ribosomal\_peptide\_synthetase\_modules\_and\_related\_proteins | BGC0000330 | Cupriachelin\_biosynthetic\_gene\_cluster | 26 | 54.4613350958 | 219 | 2.3e-56 |
| ACG60772.1 | NRPS(C/A/PCP/Cy/A/PCP/Cy) | BGC0001058 | Zorbamycin\_biosynthetic\_gene\_cluster | 24 | 85.9881031064 | 218 | 4e-56 |
| AIG79224.1 | Hypothetical\_protein | BGC0000419 | Ristomycin\_A\_biosynthetic\_gene\_cluster | 36 | 27.0984798414 | 218 | 4e-56 |
| AAT28740.1 | FUSS | BGC0000064 | Fusarin\_biosynthetic\_gene\_cluster | 24 | 66.0938532716 | 218 | 5.2e-56 |
| CAA11773.1 | PCZA361.18 | BGC0000322\_c2 | Chloroeremomycin\_biosynthetic\_gene\_cluster | 34 | 27.6933245208 | 218 | 6.7e-56 |
| CAL80821.1 | sylD-like\_NRPS/PKS | BGC0000997 | Glidobactin\_biosynthetic\_gene\_cluster | 27 | 57.2372769332 | 217 | 8.8e-56 |
| EJK79843.1 | amino\_acid\_adenylation\_enzyme/thioester\_reductase\_family\_protein | BGC0000436 | Syringolin\_A\_biosynthetic\_gene\_cluster | 26 | 66.0938532716 | 216 | 2.6e-55 |
| AGE11898.1 | nonribosomal\_peptide\_synthetase | BGC0000366 | Gobichelin\_biosynthetic\_gene\_cluster | 26 | 65.3007270324 | 216 | 2.6e-55 |
| AAN32981.1 | BarG | BGC0000962 | Barbamide\_biosynthetic\_gene\_cluster | 25 | 60.6741573034 | 216 | 2.6e-55 |
| CBG75492.1 | putative\_NRPS/siderophore\_biosynthesis\_protein | BGC0000423 | Scabichelin\_biosynthetic\_gene\_cluster | 30 | 33.9722405816 | 215 | 3.3e-55 |
| CCJ67646.1 | JagB | BGC0001127 | Jagaricin\_biosynthetic\_gene\_cluster | 27 | 60.2775941837 | 215 | 5.7e-55 |
| ABR67744.1 | CmnA | BGC0000316 | Capreomycin\_biosynthetic\_gene\_cluster | 26 | 54.990085922 | 214 | 7.5e-55 |
| ADI59531.1 | CorI | BGC0001091 | Corallopyronin\_biosynthetic\_gene\_cluster | 28 | 44.0185062789 | 214 | 7.5e-55 |
| WP\_051700105.1 | non-ribosomal\_peptide\_synthetase | BGC0001368 | Tambromycin\_biosynthetic\_gene\_cluster | 26 | 60.1454064772 | 214 | 7.5e-55 |
| AAF86395.1 | FkbP | BGC0000994 | FK520\_biosynthetic\_gene\_cluster | 24 | 80.2379378718 | 214 | 9.7e-55 |
| EFL06864.1 | non-ribosomal\_peptide\_synthetase | BGC0000300 | Amychelin\_biosynthetic\_gene\_cluster | 26 | 61.2690019828 | 214 | 9.7e-55 |
| ADH04680.1 | hybrid\_polyketide\_synthase/non-ribosomal\_peptide\_synthetase | BGC0001344 | Tubulysin\_biosynthetic\_gene\_cluster | 29 | 38.5327164574 | 213 | 2.2e-54 |
| ACZ55944.1 | non-ribosomal\_peptide\_synthetase | BGC0000302 | Anabaenopeptin\_biosynthetic\_gene\_cluster | 24 | 56.1797752809 | 213 | 2.2e-54 |
| CCP45168.1 | Peptide\_synthetase\_MbtE\_(peptide\_synthase) | BGC0001021 | Mycobactin\_biosynthetic\_gene\_cluster | 26 | 55.1883674818 | 212 | 4.8e-54 |
| AAN65224.1 | peptide\_synthetase-like\_protein | BGC0000832 | Clorobiocin\_biosynthetic\_gene\_cluster | 30 | 30.5353602115 | 211 | 6.3e-54 |
| AAN85493.1 | nonribosomal\_peptide\_synthetase | BGC0001101 | Leinamycin\_biosynthetic\_gene\_cluster | 25 | 64.5076007931 | 211 | 8.2e-54 |
| CAD29797.1 | peptide\_synthetase | BGC0001015 | Microcystin\_biosynthetic\_gene\_cluster | 31 | 33.6417713153 | 210 | 1.1e-53 |
| ABR67749.1 | CmnF | BGC0000316 | Capreomycin\_biosynthetic\_gene\_cluster | 29 | 43.4236615995 | 210 | 1.4e-53 |
| ADA82585.1 | hybrid\_trans-AT\_polyketide\_synthase\_-\_nonribosomal\_peptide\_synthetase | BGC0001110 | Psymberin\_biosynthetic\_gene\_cluster | 22 | 63.9127561137 | 210 | 1.8e-53 |
| ABC34305.1 | peptide\_synthetase,\_putative | BGC0000961 | Bactobolin\_biosynthetic\_gene\_cluster | 24 | 79.0482485129 | 210 | 1.8e-53 |
| AEF16059.1 | non-ribosomal\_peptide\_synthetase | BGC0000953 | Amicetin\_biosynthetic\_gene\_cluster | 27 | 44.9438202247 | 209 | 3.1e-53 |
| ALG65314.1 | Cal22 | BGC0001297 | WS9326\_biosynthetic\_gene\_cluster | 26 | 47.2571050892 | 208 | 4.1e-53 |
| CDG12864.1 | non-ribosomal\_peptide\_synthetase | BGC0001415 | Althiomycin\_biosynthetic\_gene\_cluster | 23 | 68.8697951091 | 208 | 4.1e-53 |
| CAJ96471.1 | Non-ribosomal\_peptide\_synthetase | BGC0000330 | Cupriachelin\_biosynthetic\_gene\_cluster | 30 | 39.4580304032 | 208 | 4.1e-53 |
| ABC34483.1 | nonribosomal\_peptide\_synthetase,\_putative | BGC0000961 | Bactobolin\_biosynthetic\_gene\_cluster | 24 | 66.0938532716 | 208 | 4.1e-53 |
| CAD32904.2 | non-ribosomal\_peptide\_synthetase\_A | BGC0000354 | Friulimicin\_biosynthetic\_gene\_cluster | 26 | 44.4811632518 | 208 | 5.3e-53 |
| CAE52339.1 | Polyketide\_non-ribosomal\_peptide\_synthase | BGC0001088 | Albicidin\_biosynthetic\_gene\_cluster | 23 | 91.9365499009 | 207 | 9.1e-53 |
| AAZ55900.1 | non-ribosomal\_peptide\_synthase:Amino\_acid\_adenylation | BGC0000359 | Fuscachelin\_biosynthetic\_gene\_cluster | 31 | 40.9120951751 | 207 | 1.2e-52 |
| AEA30272.1 | peptide\_synthetase | BGC0000429 | Skyllamycin\_biosynthetic\_gene\_cluster | 28 | 54.6596166557 | 205 | 4.5e-52 |
| AAL33756.1 | putative\_non-ribosomal\_peptide\_synthetase | BGC0000421 | Safracin\_biosynthetic\_gene\_cluster | 29 | 43.8202247191 | 205 | 5.9e-52 |
| AAW03326.1 | CtaC | BGC0000982 | Cystothiazole\_A\_biosynthetic\_gene\_cluster | 24 | 58.2947785856 | 204 | 7.7e-52 |
| ABI22133.1 | putative\_non-ribosomal\_peptide\_synthetase | BGC0000422 | Saframycin\_A\_biosynthetic\_gene\_cluster | 29 | 47.3231989425 | 204 | 1e-51 |
| CUI25728.1 | Nonribosomal\_peptide\_synthase | BGC0001355 | U-68204\_biosynthetic\_gene\_cluster | 28 | 42.1678783873 | 204 | 1e-51 |
| AAG29780.1 | acyl-CoA\_synthetase | BGC0000833 | Coumermycin\_A1\_biosynthetic\_gene\_cluster | 31 | 30.7336417713 | 204 | 1e-51 |
| AAF19811.1 | MtaC | BGC0001024 | Myxothiazol\_biosynthetic\_gene\_cluster | 29 | 39.9206873761 | 203 | 2.2e-51 |
| ABV56604.1 | adenylation\_domain\_protein | BGC0000378 | Kutznerides\_biosynthetic\_gene\_cluster | 28 | 41.3747521481 | 202 | 2.9e-51 |
| CAD70195.1 | non-ribosomal\_peptide\_synthetase | BGC0001047 | Syringolin\_A\_biosynthetic\_gene\_cluster | 24 | 66.0277594184 | 202 | 3.8e-51 |
| EOY45602.1 | non-ribosomal\_peptide\_synthetase | BGC0001168 | Livipeptin\_biosynthetic\_gene\_cluster | 30 | 38.4666226041 | 201 | 5e-51 |
| CBJ89761.1 | Non-ribosomal\_peptide\_synthase\_involved\_in\_xenocoumacin\_synthesis | BGC0001054 | Xenocoumacin\_biosynthetic\_gene\_cluster | 27 | 40.9120951751 | 201 | 6.5e-51 |
| AAN85501.1 | nonribosomal\_peptide\_synthetase | BGC0001101 | Leinamycin\_biosynthetic\_gene\_cluster | 25 | 57.9643093192 | 201 | 6.5e-51 |
| AHB38511.1 | EpnJ | BGC0000345 | Eponemycin\_biosynthetic\_gene\_cluster | 29 | 40.6477197621 | 201 | 6.5e-51 |
| AEE88284.1 | CurF | BGC0000976 | Curacin\_biosynthetic\_gene\_cluster | 24 | 62.5908790482 | 201 | 6.5e-51 |
| AAT70101.1 | CurF | BGC0001165 | Curacin\_biosynthetic\_gene\_cluster | 24 | 62.5908790482 | 201 | 6.5e-51 |
| EWS95124.1 | hypothetical\_protein | BGC0000306 | Arylomycin\_biosynthetic\_gene\_cluster | 32 | 30.7336417713 | 200 | 1.1e-50 |
| AFK57215.1 | DidD | BGC0000985 | Didemnin\_biosynthetic\_gene\_cluster | 28 | 46.46397885 | 200 | 1.5e-50 |
| AGN74898.1 | nonribosomal\_peptide\_synthetase | BGC0000459 | Griseoviridin\_/\_viridogrisein\_biosynthetic\_gene\_cluster | 31 | 34.5009914078 | 200 | 1.5e-50 |
| ADL64235.1 | aureusimine\_non-ribosomal\_peptide\_synthetase | BGC0000308 | Aureusimine\_biosynthetic\_gene\_cluster | 22 | 71.5796430932 | 200 | 1.5e-50 |
| ADN26252.1 | peptide\_synthetase | BGC0000951 | Pacidamycin\_biosynthetic\_gene\_cluster | 26 | 43.4236615995 | 200 | 1.9e-50 |
| BAH04161.1 | putative\_non-ribosomal\_peptide\_synthetase | BGC0000450 | Triostin\_A\_biosynthetic\_gene\_cluster | 26 | 57.17118308 | 199 | 2.5e-50 |
| AGC09519.1 | AMP-dependent\_synthetase/ligase | BGC0001183 | Lobophorin\_biosynthetic\_gene\_cluster | 27 | 40.383344349 | 199 | 2.5e-50 |
| EFL02193.1 | amino\_acid\_adenylation\_domain-containing\_protein | BGC0000996 | Frontalamides\_biosynthetic\_gene\_cluster | 24 | 61.1368142763 | 199 | 3.2e-50 |
| AKP45396.1 | CysH | BGC0001413 | Cystobactamide\_biosynthetic\_gene\_cluster | 33 | 27.9576999339 | 199 | 3.2e-50 |
| CUI25745.1 | Nonribosomal\_peptide\_synthase | BGC0001354 | U-68204\_biosynthetic\_gene\_cluster | 27 | 41.5730337079 | 199 | 3.2e-50 |
| ADY76667.1 | non-ribosomal\_peptide\_synthetase | BGC0000950 | Napsamycin\_biosynthetic\_gene\_cluster | 26 | 43.8202247191 | 199 | 3.2e-50 |
| ABC39418.1 | dihydroaeruginoic\_acid\_synthetase | BGC0000964 | Burkholdac\_biosynthetic\_gene\_cluster | 27 | 45.2742894911 | 198 | 4.2e-50 |
| CAE52342.1 | non-ribosomal\_peptide\_synthase | BGC0001088 | Albicidin\_biosynthetic\_gene\_cluster | 32 | 28.6186384666 | 198 | 4.2e-50 |
| AKA54626.1 | NRPS | BGC0001216 | Splenocin\_biosynthetic\_gene\_cluster | 28 | 40.5816259088 | 198 | 4.2e-50 |
| ALJ49909.1 | TlmI | BGC0001237 | Thiolactomycin\_biosynthetic\_gene\_cluster | 29 | 35.8889623265 | 197 | 9.4e-50 |
| AMK48225.1 | nonribosomal\_peptide\_synthetase | BGC0001351 | Cahuitamycins\_biosynthetic\_gene\_cluster | 30 | 39.5902181097 | 197 | 1.2e-49 |
| CBL93718.1 | NRPS\_didomain\_PCP-C | BGC0000360 | GE81112\_biosynthetic\_gene\_cluster | 27 | 46.3317911434 | 197 | 1.2e-49 |
| KPN90374.1 | NunB1 | BGC0001416 | Nunapeptin\_/\_nunamycin\_biosynthetic\_gene\_cluster | 34 | 28.3542630535 | 196 | 2.1e-49 |
| CCP45171.1 | Phenyloxazoline\_synthase\_MbtB\_(phenyloxazoline\_synthetase) | BGC0001021 | Mycobactin\_biosynthetic\_gene\_cluster | 25 | 57.898215466 | 196 | 2.7e-49 |
| AFK57219.1 | DidH | BGC0000985 | Didemnin\_biosynthetic\_gene\_cluster | 24 | 72.8354263054 | 196 | 2.7e-49 |
| ADN43685.1 | DmbS | BGC0001136 | Desmethylbassianin\_biosynthetic\_gene\_cluster | 24 | 68.5393258427 | 196 | 2.7e-49 |
| ABR67750.1 | CmnG | BGC0000316 | Capreomycin\_biosynthetic\_gene\_cluster | 30 | 41.3747521481 | 196 | 2.7e-49 |
| CAC17499.1 | putative\_non-ribosomal\_peptide\_synthase | BGC0000324 | Coelibactin\_biosynthetic\_gene\_cluster | 28 | 48.7111698612 | 196 | 2.7e-49 |
| BAF50720.1 | hybrid\_non\_ribosomal\_peptide\_synthetase-polyketide\_synthase | BGC0001116 | Virginiamycin\_biosynthetic\_gene\_cluster | 28 | 44.3489755453 | 195 | 3.6e-49 |
| ACM79806.1 | ZmaB | BGC0001059 | Zwittermycin\_A\_biosynthetic\_gene\_cluster | 24 | 46.8605419696 | 195 | 4.7e-49 |
| AHZ34243.1 | CipF | BGC0001389 | Cichopeptin\_biosynthetic\_gene\_cluster | 27 | 43.2253800397 | 195 | 6.1e-49 |
| CAD89774.1 | MelC\_protein | BGC0001009 | Melithiazol\_biosynthetic\_gene\_cluster | 24 | 59.7488433576 | 193 | 1.8e-48 |
| CCC55921.1 | non-ribosomal\_peptide\_synthetase/polyketide\_synthase\_hybrid\_protein | BGC0000973 | Collismycin\_A\_biosynthetic\_gene\_cluster | 28 | 41.2425644415 | 193 | 1.8e-48 |
| BAP16689.1 | nonribosomal\_peptide\_synthetase | BGC0000376 | JBIR-34,\_JBIR-35\_biosynthetic\_gene\_cluster | 25 | 67.2835426305 | 193 | 1.8e-48 |
| AOA33121.1 | nonribosomal\_peptide\_synthetase | BGC0001346 | Bananamides\_biosynthetic\_gene\_cluster | 32 | 30.3370786517 | 193 | 2.3e-48 |
| CAL69889.1 | RhiB\_protein | BGC0001112 | Rhizoxins\_biosynthetic\_gene\_cluster | 27 | 38.4005287508 | 192 | 3e-48 |
| AEA29644.1 | putative\_nonribosomal\_peptide\_synthetase\_and\_kinurenine\_monooxygenase | BGC0000409 | Porothramycin\_biosynthetic\_gene\_cluster | 25 | 59.0879048249 | 192 | 4e-48 |
| AAN85506.1 | nonribosomal\_peptide\_synthetase | BGC0001101 | Leinamycin\_biosynthetic\_gene\_cluster | 27 | 46.2656972902 | 191 | 5.2e-48 |
| BAE98156.1 | putative\_non-ribosomal\_peptide\_synthetase | BGC0000339 | Echinomycin\_biosynthetic\_gene\_cluster | 26 | 59.7488433576 | 190 | 1.2e-47 |
| AEC14349.1 | nonribosomal\_peptide\_synthetase | BGC0000377 | Koranimine\_biosynthetic\_gene\_cluster | 27 | 39.3919365499 | 190 | 1.2e-47 |
| CUI25672.1 | Thiolactomycin\_Hybrid\_PKS-NRPS\_system | BGC0001353 | Thiolactomycin\_biosynthetic\_gene\_cluster | 26 | 45.736946464 | 190 | 1.5e-47 |
| CCA29203.1 | non-ribosomal\_peptide\_synthetase/polyketide\_synthase | BGC0000955 | Althiomycin\_biosynthetic\_gene\_cluster | 26 | 49.4382022472 | 190 | 1.5e-47 |
| WP\_051700151.1 | hypothetical\_protein | BGC0001368 | Tambromycin\_biosynthetic\_gene\_cluster | 29 | 39.7884996695 | 190 | 1.5e-47 |
| AFD30954.1 | CrmA | BGC0000966 | Caerulomycin\_A\_biosynthetic\_gene\_cluster | 28 | 40.5155320555 | 190 | 1.5e-47 |
| ABG94125.1 | non-ribosomal\_peptide\_synthetase | BGC0000417 | Rhodochelin\_biosynthetic\_gene\_cluster | 29 | 31.2623925975 | 189 | 3.4e-47 |
| WP\_028678148.1 | non-ribosomal\_peptide\_synthetase | BGC0001228 | Retimycin\_biosynthetic\_gene\_cluster | 26 | 53.7343027098 | 188 | 4.4e-47 |
| CAO91861.1 | putative\_PKS-NRPS\_protein | BGC0000968 | Chaetoglobosin\_biosynthetic\_gene\_cluster | 29 | 42.6966292135 | 188 | 4.4e-47 |
| BAF50727.1 | hybrid\_polyketide\_synthase-non\_ribosomal\_peptide\_synthetase | BGC0001116 | Virginiamycin\_biosynthetic\_gene\_cluster | 29 | 33.7078651685 | 188 | 4.4e-47 |
| AEC14346.1 | nonribosomal\_peptide\_synthetase | BGC0000377 | Koranimine\_biosynthetic\_gene\_cluster | 26 | 42.6966292135 | 188 | 4.4e-47 |
| CAE52334.1 | non-ribosomal\_peptide\_synthase | BGC0001088 | Albicidin\_biosynthetic\_gene\_cluster | 25 | 53.1394580304 | 188 | 5.7e-47 |
| AGE11891.1 | nonribosomal\_peptide\_synthetase | BGC0000366 | Gobichelin\_biosynthetic\_gene\_cluster | 28 | 41.837409121 | 188 | 5.7e-47 |
| AAY37650.1 | Amino\_acid\_adenylation | BGC0000437 | Syringomycin\_biosynthetic\_gene\_cluster | 32 | 28.3542630535 | 187 | 9.8e-47 |
| ALJ49920.1 | TtmI | BGC0001236 | Thiotetroamide\_biosynthetic\_gene\_cluster | 28 | 40.383344349 | 187 | 9.8e-47 |
| BAO98812.1 | putative\_nonribosomal\_peptide\_synthetase | BGC0001002 | Jawsamycin\_biosynthetic\_gene\_cluster | 24 | 57.898215466 | 187 | 9.8e-47 |
| CAF05649.1 | TubD\_protein | BGC0001053 | Tubulysin\_biosynthetic\_gene\_cluster | 27 | 40.6477197621 | 186 | 2.2e-46 |
| AGN74876.1 | nonribosomal\_peptide\_synthetase | BGC0000459 | Griseoviridin\_/\_viridogrisein\_biosynthetic\_gene\_cluster | 32 | 30.0727032386 | 186 | 2.2e-46 |
| AAP92497.1 | nonribosomal\_peptide\_synthetase | BGC0000458 | Viomycin\_biosynthetic\_gene\_cluster | 26 | 56.1136814276 | 186 | 2.8e-46 |
| CBW45749.1 | hybrid\_PKS/NRPS\_(PKSI) | BGC0000952\_c3 | Pristinamycin\_biosynthetic\_gene\_cluster | 27 | 41.5069398546 | 186 | 2.8e-46 |
| ADN26251.1 | ATP-dependent\_adenylase | BGC0000951 | Pacidamycin\_biosynthetic\_gene\_cluster | 29 | 33.1130204891 | 186 | 2.8e-46 |
| AKC54422.1 | fumosorinone\_biosynthesis\_polyketide\_synthase | BGC0001218 | Fumosorinone\_biosynthetic\_gene\_cluster | 29 | 42.2339722406 | 185 | 3.7e-46 |
| AFR69331.1 | nonribosomal\_peptide\_synthetase\_SpiA | BGC0001045 | Spiruchostatin\_biosynthetic\_gene\_cluster | 27 | 40.7138136153 | 185 | 6.3e-46 |
| EAU36744.1 | predicted\_protein | BGC0000292 | Acetylaranotin\_biosynthetic\_gene\_cluster | 28 | 40.3172504957 | 185 | 6.3e-46 |
| BAI63289.1 | putative\_non-ribosomal\_peptide\_synthetase | BGC0000434 | SW-163\_biosynthetic\_gene\_cluster | 24 | 53.8664904164 | 185 | 6.3e-46 |
| AGN74895.1 | nonribosomal\_peptide\_synthetase/polyketide\_synthase\_hybrid\_protein | BGC0000459 | Griseoviridin\_/\_viridogrisein\_biosynthetic\_gene\_cluster | 29 | 38.8631857237 | 184 | 8.3e-46 |
| ACR33075.1 | proline\_adenylation\_protein | BGC0000017 | Anatoxin\_biosynthetic\_gene\_cluster | 29 | 35.6906807667 | 184 | 1.1e-45 |
| CBZ42140.1 | non-ribosomal\_peptide\_synthetase | BGC0001117 | Himastatin\_biosynthetic\_gene\_cluster | 27 | 41.1764705882 | 184 | 1.1e-45 |
| CAN89664.1 | putative\_amino\_acid\_adenylating\_enzyme | BGC0001070 | Kirromycin\_biosynthetic\_gene\_cluster | 31 | 34.6331791143 | 184 | 1.1e-45 |
| AET98905.1 | putative\_non-ribosomal\_peptide\_synthetase | BGC0000415 | Quinomycin\_biosynthetic\_gene\_cluster | 25 | 53.9325842697 | 183 | 2.4e-45 |
| AHD05616.1 | putative\_non-ribosomal\_peptide\_ligase\_domain\_protein | BGC0001033 | Paenilamicin\_biosynthetic\_gene\_cluster | 30 | 36.5499008592 | 183 | 2.4e-45 |
| ABG00046.1 | non-ribosomal\_peptide\_synthetase | BGC0000387 | Mangotoxin\_biosynthetic\_gene\_cluster | 27 | 41.5730337079 | 182 | 4.1e-45 |
| EFG10344.1 | Non-ribosomal\_peptide\_synthetase | BGC0000373 | Holomycin\_biosynthetic\_gene\_cluster | 27 | 40.8460013219 | 181 | 7e-45 |
| AAT12283.1 | LtxA | BGC0000384 | Lyngbyatoxin\_biosynthetic\_gene\_cluster | 30 | 30.667547918 | 181 | 7e-45 |
| CUI25701.1 | amino\_acid\_adenylation\_domain-containing\_protein\_potentially\_involved\_in\_thiotetronic\_acid\_synthesis | BGC0001352 | Thiotetronate\_Tu\_3010\_biosynthetic\_gene\_cluster | 27 | 40.5816259088 | 181 | 9.1e-45 |
| AAG02364.1 | peptide\_synthetase\_NRPS2-1 | BGC0000963 | Bleomycin\_biosynthetic\_gene\_cluster | 27 | 42.2339722406 | 181 | 9.1e-45 |
| AAZ23077.1 | peptide\_synthetase | BGC0000291 | A54145\_biosynthetic\_gene\_cluster | 28 | 45.3403833443 | 180 | 1.6e-44 |
| AEA29643.1 | putative\_nonribosomal\_peptide\_synthetase | BGC0000409 | Porothramycin\_biosynthetic\_gene\_cluster | 27 | 41.4408460013 | 180 | 2e-44 |
| AHB38515.1 | EpnG | BGC0000345 | Eponemycin\_biosynthetic\_gene\_cluster | 28 | 40.383344349 | 180 | 2e-44 |
| AAP92505.1 | nonribosomal\_peptide\_synthetase | BGC0000458 | Viomycin\_biosynthetic\_gene\_cluster | 27 | 41.9035029742 | 179 | 2.7e-44 |
| CAJ34375.1 | NRPS | BGC0000445 | Thiocoraline\_biosynthetic\_gene\_cluster | 24 | 53.3377395902 | 178 | 4.5e-44 |
| AAY42397.1 | HctE | BGC0001000 | Hectochlorin\_biosynthetic\_gene\_cluster | 27 | 40.383344349 | 178 | 7.7e-44 |
| AJW76709.1 | DsaG | BGC0001196 | Desotamide\_biosynthetic\_gene\_cluster | 30 | 33.7078651685 | 177 | 1e-43 |
| AAW03327.1 | CtaD | BGC0000982 | Cystothiazole\_A\_biosynthetic\_gene\_cluster | 32 | 28.222075347 | 177 | 1.3e-43 |
| EJK79842.1 | amino\_acid\_adenylation\_enzyme/thioester\_reductase\_family\_protein | BGC0000436 | Syringolin\_A\_biosynthetic\_gene\_cluster | 25 | 47.6536682089 | 177 | 1.3e-43 |
| CAD70194.1 | non-ribosomal\_peptide\_synthetase | BGC0001047 | Syringolin\_A\_biosynthetic\_gene\_cluster | 24 | 46.5961665565 | 177 | 1.3e-43 |
| CAQ18831.1 | nonribosomal\_polypetide\_synthetase | BGC0000954 | Ajudazol\_biosynthetic\_gene\_cluster | 28 | 31.3284864508 | 176 | 1.7e-43 |
| AAY42398.1 | HctF | BGC0001000 | Hectochlorin\_biosynthetic\_gene\_cluster | 27 | 37.0125578321 | 176 | 1.7e-43 |
| CAC17500.1 | putative\_non-ribosomal\_peptide\_synthase | BGC0000324 | Coelibactin\_biosynthetic\_gene\_cluster | 31 | 28.6186384666 | 176 | 2.9e-43 |
| CBW45740.1 | hybrid\_NRPS/PKS\_(PKSIV) | BGC0000952\_c3 | Pristinamycin\_biosynthetic\_gene\_cluster | 25 | 43.4897554527 | 175 | 5e-43 |
| CBK62746.1 |  | BGC0001115 | Thiomarinol\_biosynthetic\_gene\_cluster | 23 | 60.343688037 | 175 | 6.6e-43 |
| AFK57214.1 | DidC | BGC0000985 | Didemnin\_biosynthetic\_gene\_cluster | 29 | 41.110376735 | 175 | 6.6e-43 |
| AHD05621.1 | non-ribosomal\_peptide\_ligase\_domain\_protein | BGC0001033 | Paenilamicin\_biosynthetic\_gene\_cluster | 30 | 31.5928618638 | 174 | 8.6e-43 |
| ABP57745.1 | DepA | BGC0000993 | FK228\_biosynthetic\_gene\_cluster | 28 | 39.1936549901 | 173 | 1.5e-42 |
| AAN85522.1 | hybrid\_nonribosomal\_peptide\_synthetase\_/\_polyketide\_synthase | BGC0001101 | Leinamycin\_biosynthetic\_gene\_cluster | 23 | 65.7633840053 | 173 | 1.5e-42 |
| BAD55613.1 | putative\_non-ribosomal\_peptide\_synthetase | BGC0001027 | Nocobactin\_NA\_biosynthetic\_gene\_cluster | 28 | 40.9781890284 | 173 | 1.9e-42 |
| ABL74945.1 | TlmIV | BGC0001048 | Tallysomycin\_biosynthetic\_gene\_cluster | 25 | 58.162590879 | 172 | 3.3e-42 |
| ABD65960.1 | nonribosomal\_peptide\_synthetase | BGC0000341 | Enduracidin\_biosynthetic\_gene\_cluster | 30 | 28.4203569068 | 172 | 3.3e-42 |
| CBL93716.1 | NRPS\_A-domain | BGC0000360 | GE81112\_biosynthetic\_gene\_cluster | 28 | 34.8975545274 | 171 | 7.2e-42 |
| CAJ76286.1 | putative\_non-ribosomal\_peptide\_synthetase | BGC0000972 | Colibactin\_biosynthetic\_gene\_cluster | 26 | 39.3258426966 | 171 | 9.5e-42 |
| AAF19812.1 | MtaD | BGC0001024 | Myxothiazol\_biosynthetic\_gene\_cluster | 26 | 44.0185062789 | 170 | 1.2e-41 |
| AFV27434.1 | indigoidine\_synthase | BGC0000375 | Indigoidine\_biosynthetic\_gene\_cluster | 32 | 28.3542630535 | 170 | 1.2e-41 |
| AKA59447.1 | non-ribosomal\_peptide\_synthetase | BGC0001203 | Clarexpoxcin\_biosynthetic\_gene\_cluster | 27 | 40.6477197621 | 170 | 1.6e-41 |
| CAD15508.1 | probable\_polyketide\_synthase\_protein | BGC0001014 | Micacocidin\_biosynthetic\_gene\_cluster | 29 | 32.848645076 | 169 | 2.8e-41 |
| CAD29794.1 | peptide\_synthetase | BGC0001015 | Microcystin\_biosynthetic\_gene\_cluster | 24 | 43.2253800397 | 168 | 6.1e-41 |
| WP\_010639240.1 | gramicidin\_synthetase | BGC0000958 | Antimycin\_biosynthetic\_gene\_cluster | 30 | 33.6417713153 | 168 | 8e-41 |
| AAL33758.1 | putative\_non-ribosomal\_peptide\_synthetase | BGC0000421 | Safracin\_biosynthetic\_gene\_cluster | 24 | 51.0244547257 | 166 | 1.8e-40 |
| EAL92291.2 | nonribosomal\_peptide\_synthase\_GliP-like,\_putative | BGC0000372 | Hexadehydro-astechrome\_(HAS)\_biosynthetic\_gene\_cluster | 29 | 39.8545935228 | 165 | 5.2e-40 |
| CDG12865.1 | non-ribosomal\_peptide\_synthetase | BGC0001415 | Althiomycin\_biosynthetic\_gene\_cluster | 27 | 33.9061467284 | 163 | 1.5e-39 |
| ADZ45324.1 | non-ribosomal\_peptide\_synthetase | BGC0001020 | Muraymycin\_biosynthetic\_gene\_cluster | 23 | 64.5076007931 | 163 | 2e-39 |
| ABX71111.1 | Lct28 | BGC0000238 | Lactonamycin\_biosynthetic\_gene\_cluster | 27 | 35.2280237938 | 163 | 2e-39 |
| CAD89775.1 | MelD\_protein | BGC0001009 | Melithiazol\_biosynthetic\_gene\_cluster | 30 | 28.6847323199 | 163 | 2.6e-39 |
| ADH04660.1 | TugD | BGC0001342 | Thuggacin\_biosynthetic\_gene\_cluster | 28 | 35.4923992069 | 162 | 3.4e-39 |
| AJI44176.1 | nonribosomal\_peptide\_synthetase | BGC0001193 | Thiolutin\_biosynthetic\_gene\_cluster | 26 | 40.6477197621 | 162 | 4.4e-39 |
| CCB53267.1 | non-ribosomal\_peptide\_synthetase | BGC0001393 | Lugdunin\_biosynthetic\_gene\_cluster | 21 | 55.9814937211 | 161 | 5.7e-39 |
| BAC76476.1 | multifunctional\_polyketide-peptide\_synthase\_LkcA | BGC0001100 | Lankacidin\_biosynthetic\_gene\_cluster | 28 | 42.4983476537 | 161 | 9.8e-39 |
| ADH04641.1 | TgaC | BGC0001051 | Thuggacin\_biosynthetic\_gene\_cluster | 30 | 28.5525446134 | 161 | 9.8e-39 |
| CAF32362.1 | putative\_non-ribosomal\_peptide\_synthetase | BGC0000712 | Paromomycin\_biosynthetic\_gene\_cluster | 28 | 39.9867812293 | 160 | 2.2e-38 |
| BAL15718.1 | NRPS | BGC0000432 | Streptothricin\_biosynthetic\_gene\_cluster | 31 | 28.8830138797 | 159 | 3.7e-38 |
| AHA12086.1 | amino\_acid\_adenyltransferase | BGC0001172 | Chlorizidine\_A\_biosynthetic\_gene\_cluster | 29 | 34.9636483807 | 158 | 8.3e-38 |
| AFK57220.1 | DidI | BGC0000985 | Didemnin\_biosynthetic\_gene\_cluster | 28 | 37.2108393919 | 157 | 1.1e-37 |
| ACO94464.1 | NRPS\_adenylation\_domain\_protein | BGC0000029 | BE-14106\_biosynthetic\_gene\_cluster | 28 | 34.4348975545 | 157 | 1.1e-37 |
| ADB02873.1 | AzicV | BGC0000202 | Azicemicin\_biosynthetic\_gene\_cluster | 28 | 34.2366159947 | 156 | 2.4e-37 |
| CAJ76289.1 | putative\_hybrid\_non-ribosomal\_peptide-polyketide\_synthetase | BGC0000972 | Colibactin\_biosynthetic\_gene\_cluster | 28 | 29.8083278255 | 156 | 3.1e-37 |
| ALV82447.1 | putative\_non-ribosomal\_peptide\_synthetase | BGC0001382 | Svaricin\_biosynthetic\_gene\_cluster | 25 | 55.9153998678 | 155 | 4.1e-37 |
| AAG05690.1 | AmbE | BGC0000287 | 2-amino-4-methoxy-trans-3-butenoic\_acid\_biosynthetic\_gene\_cluster | 29 | 30.8658294779 | 155 | 5.4e-37 |
| BAP16684.1 | nonribosomal\_peptide\_synthetase | BGC0000376 | JBIR-34,\_JBIR-35\_biosynthetic\_gene\_cluster | 31 | 26.6358228685 | 154 | 1.2e-36 |
| CAJ87590.1 | putative\_peptide\_synthase | BGC0001055 | Yersiniabactin\_biosynthetic\_gene\_cluster | 27 | 32.9808327826 | 153 | 2e-36 |
| ACO94489.1 | putative\_AMP-dependent\_acyl-CoA\_synthetase/ligase | BGC0000097 | ML-449\_biosynthetic\_gene\_cluster | 29 | 35.8228684732 | 153 | 2e-36 |
| CCA29202.1 | non-ribosomal\_peptide\_synthetase | BGC0000955 | Althiomycin\_biosynthetic\_gene\_cluster | 29 | 30.9319233311 | 152 | 3.5e-36 |
| ABX60161.1 | mixed\_NRPS/PKS | BGC0000978 | Cylindrospermopsin\_biosynthetic\_gene\_cluster | 30 | 31.8572372769 | 152 | 3.5e-36 |
| AAV66110.2 | fusaridione\_A\_synthetase | BGC0000992 | Fusaridione\_A\_biosynthetic\_gene\_cluster | 25 | 46.0674157303 | 151 | 7.8e-36 |
| ADF88262.1 | mixed\_nonribosomal\_peptide\_synthetase/\_polyketide\_synthase | BGC0000979 | Cylindrospermopsin\_biosynthetic\_gene\_cluster | 30 | 31.8572372769 | 151 | 7.8e-36 |
| ADF88279.1 | mixed\_NRPS/PKS | BGC0000981 | Cylindrospermopsin\_biosynthetic\_gene\_cluster | 30 | 31.8572372769 | 151 | 7.8e-36 |
| ADF88265.1 | mixed\_nonribosomal\_peptide\_synthetase/\_polyketide\_synthase | BGC0000980 | Cylindrospermopsin\_biosynthetic\_gene\_cluster | 30 | 31.8572372769 | 150 | 1.3e-35 |
| AAO07755.1 | Non-ribosomal\_peptide\_synthetase\_module,\_siderophore\_biosynthesis\_vulnibactin-specific | BGC0000460 | Vulnibactin\_biosynthetic\_gene\_cluster | 26 | 33.1791143424 | 149 | 2.9e-35 |
| AAC83657.1 | pyochelin\_synthetase | BGC0000412 | Pyochelin\_biosynthetic\_gene\_cluster | 28 | 31.7250495704 | 149 | 3.8e-35 |
| ACO94492.1 | NRPS\_adenylation\_domain\_protein | BGC0000097 | ML-449\_biosynthetic\_gene\_cluster | 31 | 28.7508261732 | 147 | 1.5e-34 |
| AKD43757.1 | HerL | BGC0001349 | Heronamide\_biosynthetic\_gene\_cluster | 28 | 34.8314606742 | 146 | 1.9e-34 |
| NP\_231840.1 | nonribosomal\_peptide\_synthetase\_VibF | BGC0000945\_c2 | Vibriobactin\_biosynthetic\_gene\_cluster | 25 | 40.7138136153 | 145 | 4.3e-34 |
| BAO66533.1 | nonribosomal\_peptide\_synthase | BGC0000042 | Cremimycin\_biosynthetic\_gene\_cluster | 28 | 34.8975545274 | 145 | 5.6e-34 |
| EAW09117.1 | hybrid\_NRPS/PKS\_enzyme,\_putative | BGC0000983 | Cytochalasin\_biosynthetic\_gene\_cluster | 25 | 41.7713152677 | 144 | 9.5e-34 |
| AAN85530.1 | nonribosomal\_peptide\_synthetase,\_discrete\_adenylation\_domain | BGC0001101 | Leinamycin\_biosynthetic\_gene\_cluster | 28 | 35.6245869134 | 144 | 9.5e-34 |
| AGI61652.1 | long\_chain\_fatty\_acid\_CoA\_ligase | BGC0000095\_c2 | Micromonolactam\_biosynthetic\_gene\_cluster | 29 | 35.6906807667 | 141 | 6.1e-33 |
| AEH41789.1 | HrmK | BGC0000374 | Hormaomycin\_biosynthetic\_gene\_cluster | 26 | 35.4263053536 | 141 | 6.1e-33 |
| ABX71145.1 | Lcz28 | BGC0000237 | Lactonamycin\_Z\_biosynthetic\_gene\_cluster | 26 | 35.4923992069 | 140 | 1.4e-32 |
| CAA46313.1 | peptide\_synthetase\_PhsA | BGC0000406 | Phosphinothricin\_biosynthetic\_gene\_cluster | 26 | 41.6391275611 | 140 | 1.4e-32 |
| BAR73008.1 | putative\_ATP-dependent\_b-aminoacyl-ACP\_synthetase | BGC0001194 | Hitachimycin\_biosynthetic\_gene\_cluster | 28 | 34.6331791143 | 139 | 2.3e-32 |
| BAO66530.1 | nonribosomal\_peptide\_synthase | BGC0000042 | Cremimycin\_biosynthetic\_gene\_cluster | 27 | 35.029742234 | 139 | 4e-32 |
| ABP55216.1 | AMP-dependent\_synthetase\_and\_ligase | BGC0000142 | Salinilactam\_biosynthetic\_gene\_cluster | 28 | 35.4923992069 | 139 | 4e-32 |
| ALA09365.1 | AMP-dependent\_synthetase\_and\_ligase | BGC0001303 | Lobosamide\_biosynthetic\_gene\_cluster | 28 | 35.5584930601 | 138 | 5.2e-32 |
| BAD08370.1 | non-ribosomal\_peptide\_synthetase | BGC0000167 | Vicenistatin\_biosynthetic\_gene\_cluster | 26 | 36.9464639788 | 137 | 1.5e-31 |
| ACO94461.1 | putative\_AMP-dependent\_acyl-CoA\_synthetase/ligase | BGC0000029 | BE-14106\_biosynthetic\_gene\_cluster | 28 | 35.6906807667 | 136 | 2e-31 |
| ALA09366.1 | AMP-dependent\_synthetase\_and\_ligase | BGC0001303 | Lobosamide\_biosynthetic\_gene\_cluster | 27 | 35.4923992069 | 135 | 4.4e-31 |
| BAP34707.1 | AMP-dependent\_synthetase\_and\_ligase | BGC0000078 | Incednine\_biosynthetic\_gene\_cluster | 28 | 35.3602115003 | 135 | 5.8e-31 |
| AKD43754.1 | HerJ | BGC0001349 | Heronamide\_biosynthetic\_gene\_cluster | 28 | 33.9061467284 | 135 | 5.8e-31 |
| ABP55217.1 | AMP-dependent\_synthetase\_and\_ligase | BGC0000142 | Salinilactam\_biosynthetic\_gene\_cluster | 28 | 36.2194315929 | 134 | 7.5e-31 |
| AHA34037.1 | Bmp4 | BGC0000890 | Bromophenols\_/\_bromopyrroles\_biosynthetic\_gene\_cluster | 25 | 35.2941176471 | 134 | 7.5e-31 |
| CCB53269.1 | AMP-binding\_enzyme | BGC0001393 | Lugdunin\_biosynthetic\_gene\_cluster | 26 | 28.5525446134 | 134 | 9.8e-31 |
| XP\_001220460.1 | hypothetical\_protein | BGC0001182 | Chaetoglobosins\_biosynthetic\_gene\_cluster | 24 | 43.3575677462 | 131 | 8.3e-30 |
| AAO39104.1 | AdmJ | BGC0000956 | Andrimid\_biosynthetic\_gene\_cluster | 25 | 34.3688037013 | 129 | 2.4e-29 |
| AGI61651.1 | long\_chain\_fatty\_acid\_CoA\_ligase | BGC0000095 | Micromonolactam\_biosynthetic\_gene\_cluster | 26 | 35.4923992069 | 129 | 3.2e-29 |
| AAO39105.1 | AdmK | BGC0000956 | Andrimid\_biosynthetic\_gene\_cluster | 25 | 41.5069398546 | 128 | 5.4e-29 |
| ADN26257.1 | ATP-dependent\_adenylase | BGC0000951 | Pacidamycin\_biosynthetic\_gene\_cluster | 26 | 35.029742234 | 125 | 6e-28 |
| ABB69752.1 | PlaP4 | BGC0000654 | Phenalinolactone\_biosynthetic\_gene\_cluster | 27 | 25.2478519498 | 118 | 9.5e-26 |
| CAP20364.1 | equibactin\_equibactin\_nonribosomal\_peptide\_synthase\_protein | BGC0000347 | Equibactin\_biosynthetic\_gene\_cluster | 24 | 29.4778585592 | 116 | 2.8e-25 |
| BAP34718.1 | AMP-dependent\_synthetase\_and\_ligase | BGC0000078 | Incednine\_biosynthetic\_gene\_cluster | 32 | 21.2822207535 | 109 | 4.4e-23 |
| ABS90473.1 | NRPS | BGC0001106 | Oxazolomycin\_biosynthetic\_gene\_cluster | 26 | 33.6417713153 | 104 | 1.4e-21 |
| CBW45681.1 | putative\_amino\_acid\_adenyltransferase | BGC0000952\_c2 | Pristinamycin\_biosynthetic\_gene\_cluster | 28 | 24.7851949769 | 100 | 1.6e-20 |
| ADQ55476.1 | non-ribosomal\_peptide\_synthase\_dimodule | BGC0000350 | ET-743\_biosynthetic\_gene\_cluster | 25 | 21.4805023133 | 89 | 2.8e-17 |
| AAF00961.1 | McyB | BGC0001017 | Microcystin\_biosynthetic\_gene\_cluster | 25 | 27.9576999339 | 86 | 3.1e-16 |
| EFY94493.1 | nonribosomal\_peptide\_synthase\_GliP-like,\_putative | BGC0000337 | Destruxins\_biosynthetic\_gene\_cluster | 25 | 28.4203569068 | 84 | 1.5e-15 |
| ABM21572.1 | CrpD | BGC0000975 | Cryptophycin\_biosynthetic\_gene\_cluster | 25 | 20.4230006609 | 84 | 1.5e-15 |
| ACM68690.1 | AerG1 | BGC0000298 | Aeruginosin\_biosynthetic\_gene\_cluster | 22 | 27.8255122274 | 79 | 3.7e-14 |
| ADI58632.1 | AsuB1 | BGC0000187 | Asukamycin\_biosynthetic\_gene\_cluster | 25 | 30.667547918 | 77 | 1.1e-13 |
| AAO62587.1 | peptide\_sythetase | BGC0001016 | Microcystin\_biosynthetic\_gene\_cluster | 22 | 28.0898876404 | 72 | 3.5e-12 |
| AAF00957.1 | McyG | BGC0001017 | Microcystin\_biosynthetic\_gene\_cluster | 23 | 25.9748843358 | 63 | 2.1e-09 |
